# Transcription Factor Repertoire of Necrotrophic Fungal Phytopathogen *Ascochyta rabiei*: Predominance of MYB Transcription Factors As Potential Regulators of Secretome

**DOI:** 10.3389/fpls.2017.01037

**Published:** 2017-06-14

**Authors:** Sandhya Verma, Rajesh K. Gazara, Praveen K. Verma

**Affiliations:** Plant Immunity Laboratory, National Institute of Plant Genome ResearchNew Delhi, India

**Keywords:** plant–pathogen interaction, *Ascochyta rabiei*, necrotrophic fungi, transcription factors, *cis*-acting elements, secretome

## Abstract

Transcription factors (TFs) are the key players in gene expression and their study is highly significant for shedding light on the molecular mechanisms and evolutionary history of organisms. During host–pathogen interaction, extensive reprogramming of gene expression facilitated by TFs is likely to occur in both host and pathogen. To date, the knowledge about TF repertoire in filamentous fungi is in infancy. The necrotrophic fungus *Ascochyta rabiei*, that causes destructive Ascochyta blight (AB) disease of chickpea (*Cicer arietinum*), demands more comprehensive study for better understanding of *Ascochyta*-legume pathosystem. In the present study, we performed the genome-wide identification and analysis of TFs in *A. rabiei*. Taking advantage of *A. rabiei* genome sequence, we used a bioinformatic approach to predict the TF repertoire of *A. rabiei*. For identification and classification of *A. rabiei* TFs, we designed a comprehensive pipeline using a combination of BLAST and InterProScan software. A total of 381 *A. rabiei* TFs were predicted and divided into 32 fungal specific families of TFs. The gene structure, domain organization and phylogenetic analysis of abundant families of *A. rabiei* TFs were also carried out. Comparative study of *A. rabiei* TFs with that of other necrotrophic, biotrophic, hemibiotrophic, symbiotic, and saprotrophic fungi was performed. It suggested presence of both conserved as well as unique features among them. Moreover, *cis*-acting elements on promoter sequences of earlier predicted *A. rabiei* secretome were also identified. With the help of published *A. rabiei* transcriptome data, the differential expression of TF and secretory protein coding genes was analyzed. Furthermore, comprehensive expression analysis of few selected *A. rabiei* TFs using quantitative real-time polymerase chain reaction revealed variety of expression patterns during host colonization. These genes were expressed in at least one of the time points tested post infection. Overall, this study illustrates the first genome-wide identification and analysis of TF repertoire of *A. rabiei*. This work would provide the basis for further studies to dissect role of TFs in the molecular mechanisms during *A. rabiei*–chickpea interactions.

## Introduction

A very adaptable and economical source of protein available to mankind is legumes. Apart from having high nutritional value, legumes also serve as great natural soil fertilizers. They provide nitrogen to the crops by fixing atmospheric nitrogen, thereby, decreasing the use of artificial nitrogen fertilizers. This ultimately minimizes the side effects that artificial fertilizers impose on our environment. However, like all plants, legumes are also challenged by various biotic and abiotic stresses causing major yield loss worldwide. The defense responses of plants in combating these environmental stresses are crucial for completing their lifecycle successfully.

Chickpea is one of the important legume crops grown worldwide. Its global production is estimated to be 14 million metric tons (FAO, 2014). India is the largest producer with annual production of about 10 million tons that accounts for 70% of the total world production. However, chickpea faces Ascochyta blight (AB) as a major constraint to its production. It can result in 100% crop mortality and complete yield loss (Singh et al., 1984). Due to several epidemics of AB, substantial losses have been reported from India, Pakistan, Australia, Spain (Pande et al., 2005), Canada (Chandirasekaran et al., 2009), Latin America (Kaiser et al., 2000), and United States (Kaiser et al., 1994). AB is a foliar disease caused by the necrotrophic fungus *Ascochyta rabiei* (Pass.) Labrouse [teleomorph: *Didymella rabiei* (Kovachevski) v. Arx]. This fungus belongs to dothideomycetes class of filamentous fungi. It hibernates on crop residues and seeds between seasons and readily gets transmitted through infected seeds and airborne ascospores. The conidia present in the field sometimes leads to several cycles of infection in the same season when favorable conditions prevail. *A. rabiei* penetrates the host directly through the cuticle and sometimes through stomata as well, eventually resulting in necrosis (Jayakumar et al., 2005). Despite after several pathological and molecular studies, the pathogenicity mechanisms of *A. rabiei* are still poorly understood. However in recent years, an in-depth and path-breaking research has been done in *A. rabiei* that changed many of the conventional concepts. One such example is a report where it was revealed that the solanapyrones toxins of *A. rabiei* are not required for pathogenicity (Kim et al., 2015). This suggests that necrotrophic fungi exploit the cell death machinery of the host plant for pathogenesis, which abandons the earlier concept that necrotrophic fungi solely relies on lytic enzymes for causing necrosis. Recently, the transcriptome analysis of *A. rabiei* has been carried out and a number of transcripts and putatively secreted proteins coding transcripts up-regulated during infection were identified (Fondevilla et al., 2015).

In order to solve the mystery of plant–pathogen interaction, the intricate biological mechanisms of the pathogen needs to be well addressed. Various signaling components and downstream factors play crucial role in connecting the specific pathways into an interlaced regulatory network. Among such components, transcription factors (TFs) are vital for regulating numerous mechanisms and responses. TFs are proteins that have at least one DNA-binding domain (DBD) and an activation domain (AD). The DBD binds to the sequence-specific *cis*-acting elements present in promoters of the target genes. On the other hand, the AD triggers transcription from promoter by recruiting the transcriptional machinery. Besides these, other domains might occur to facilitate homo/hetero-dimerization or the binding with co-activators or co-repressors. The DBDs of TFs are usually highly conserved and are the basis of classification of TFs into superclasses and classes (Stegmaier et al., 2004). By contrast, the ADs are far less conserved.

Knowledge about the TFs repertoire in phytopathogenic fungi is still very limited. Most of the studies have been carried out in *Saccharomyces cerevisiae* and *Schizosaccharomyces pombe* (Spitz and Furlong, 2012). With the rise in ease and availability of whole genome and transcriptome data, genome-wide identification of TFs prove to be highly beneficial to get insights of the TF repertoire present in an organism. Genome-wide search and functional identification of TFs has been carried out in the mycorrhizal fungus *Tuber melanosporum* (Montanini et al., 2011). A bioinformatics approach in combination with functional analysis in yeast and transcriptome profiling was performed to identify *T. melanosporum* TFs. Montanini et al. (2011) found that *T. melanosporum* consists of 102 homologs of previously characterized TFs, 57 homologs of hypothetical TFs, and 42 putative TFs apparently specific to *Tuber*. About one-fifth of the *in silico* predicted TFs of *T. melanosporum* were validated by yeast screen. Moreover, 29 TFs were up-regulated in ectomycorrhiza or fruiting bodies. There are other numerous studies and databases related to genome-wide search of TFs in plants (Guo et al., 2008; Mochida et al., 2010), mouse (Kanamori et al., 2004; Zheng et al., 2008), *Drosophila melanogaster* (Adryan and Teichmann, 2006), human (Lee et al., 2007; Zheng et al., 2008; Fulton et al., 2009), and rat (Zheng et al., 2008). Similarly, AnimalTFDB is a comprehensive TF database for 50 animal species ranging from *Caenorhabditis elegans* to human (Zhang et al., 2012). TRANSFAC database and its module TRANSCompel are available to study transcriptional gene regulation in eukaryotes (Matys et al., 2006). The DBD database consists of predicted TF repertoires for 930 completely sequenced genomes of eukaryotes and prokaryotes (Wilson et al., 2008). Still there are limited resources available to study TFs in case of phytopathogenic fungi.

With the available genome of *A. rabiei* (Verma et al., 2016), we have the unique prospect to identify and study a global view of putative TFs repertoire present in *A. rabiei*, for the first time. In the present study, the aim was to obtain the largest possible catalog of DBD-containing proteins which are the *bona fide* transcriptional regulators encoded by *A. rabiei* genome. The *A. rabiei* putative TF repertoire has been compared with that of several other filamentous fungi. Gene structure and phylogenetic analyses have been performed to shed light on the basic information about putative TFs of *A. rabiei*. In order to gain clues regarding expression profiles of these putative TFs and their relevance in regulation of *A. rabiei* secretome during infection, we explored the published transcriptomic profiles in the mycelium and infected chickpea leaves (Fondevilla et al., 2015). The results obtained herein gives us insight into the putative TF repertoire of *A. rabiei* and provides a significant basis for future studies on functional characterization of TFs involved in various biological processes of the phytopathogenic fungi.

## Materials and Methods

### Data Collection and Identification of *A. rabiei* Transcription Factors

The list of TFs of selected 30 fungal species was obtained from the publicly available databases: DBD release 2.0^1^ and FTFD v1.2^2^. To acquire the protein sequences of TFs, the proteomes of these fungi were downloaded from JGI. Using the protein IDs, the corresponding protein sequences of TFs were retrieved for proteomes and were used as a database. A total of 10,596 protein sequences of *A. rabiei* (Supplementary Dataset S1) were used as queries to identify all possible TFs by performing BLASTP (*e*-value cutoff ≤ 1*e*^-5^) search against TFs of 30 fungal species as database. This resulted in 1,854 hits as subject sequences. InterProScan v5.21-60.0^3^ was employed to identify TF DBDs belonging to 12 superfamilies and 37 PFAM families in fungi in the resultant 1,854 hits. A total of 392 protein sequences had DBDs and these sequences were used to perform BLASTP with FTFD at default parameters. Out of 392, 376 protein sequences returned significant hits, while 16 protein sequences did not show any hits. These 16 protein sequences were further used as queries to perform BLAST searches in NCBI and 5 of them showed significant similarity to fungal TFs in the database. Therefore, a total of 381 putative TFs were predicted.

### Gene Structure Analysis, Domain Organization, and Phylogenetic Analysis

The exon and intron structures of individual TF coding genes belonging to different families were illustrated with the help of Gene Structure Display Server v2.0^4^ (Hu et al., 2015) by aligning the cDNA sequences with the corresponding genomic DNA sequences. The functional motifs or domains of putative TF protein sequences were analyzed using CDvist^5^ (Adebali et al., 2015). For phylogenetic analysis, multiple sequence alignments of the full-length protein sequences were performed using MUSCLE v3.8.31^6^ with maxiters set at 1000. The acquired alignment was used to carry out phylogenetic analyses using Bayesian Inference (BI) implemented in MrBayes v3.2.6 (Ronquist and Huelsenbeck, 2003). The protein sequence alignments were run over 3,000,000 generations under a mixed amino acid substitution model with two independent runs and each containing four Markov Chain Monte Carlo (MCMC) chains. To estimate the posterior probabilities for each node, a sampling frequency was set at 300 iterations with MCMC left at default settings. The consensus tree was finally generated with the help of Sumt function of MrBayes. By removing the burn-in generations for each run the posterior probabilities were estimated. All the phylogenetic trees were visualized using FigTree v1.4^7^.

### *De Novo* Motif Discovery

The RSAT suite for fungi (Regulatory Sequence Analysis Tools^8^) was used to identify motifs enriched in the 1 kb promoter sequences of genes encoding secretory proteins using pleosporales as the background model and 6-, 7-, or 8-bp-long seeds on both the strands. Once we had the *cis*-regulatory elements identified across the promoter regions, annotation of *cis*-regulatory elements were carried out by scanning UniPROBE database^9^ (Newburger and Bulyk, 2009).

### Identification of Differentially Expressed Genes

RNA-seq reads available for *A. rabiei* grown “*in medium*” and “*in planta*” at time points 12, 36, and 96 hours post inoculation (hpi) were downloaded from NCBI (Fondevilla et al., 2015). Reads were mapped on the genome of *A. rabiei* using TopHat2.1.0 software (Kim et al., 2013). Transcriptional levels were estimated with Cufflinks v2.1.1 (Trapnell et al., 2013) and normalized by fragments per kilobase of transcript per million mapped reads (FPKM). The differential expression between *in medium* and *in planta* at different time points was determined by Cuffdiff v2.2.1 (Trapnell et al., 2013). The transcripts with difference of at least two-fold change along with *P*-value ≤ 0.05 were considered to be significantly differentially expressed. The *P*-value was generated by Benjamini–Hochberg correction for multiple tests running in the background of Cuffdiff v2.2.1.

### Culture Conditions and Plant Infection

Vegetative mycelia of *A. rabiei* isolate ArD2 (Indian Type Culture Collection No. 4638) were grown on potato dextrose agar (PDA; Difco Laboratories, United states) for 20 days in dark. For harvesting fungal tissue, *A. rabiei* spores were grown in potato dextrose broth (PDB; Difco Laboratories, United states) for 5 days in dark at 22°C in an incubator shaker at 120 rpm. Mycelial samples were collected, immediately frozen in liquid nitrogen and stored at -80°C. For plant infection, 21 days old chickpea seedlings (Pusa-362) were spray inoculated with *A. rabiei* spore suspensions of concentration 2 × 10^5^ spores/ml. Infected leaves and stems were harvested at 12, 24, 72, and 144 hpi with three biological replicates and stored as above.

### RNA Isolation and Quantitative Real-Time PCR

Total RNA was isolated from *A. rabiei* infected chickpea samples using the TRIzol^®^ reagent (Invitrogen, United states). The contaminating genomic DNA was removed by treating samples with RNase-free RQ1 DNase (Promega, United states) as per the manufacturer’s instruction. One microgram of total RNA primed with Oligo-dT was used for first-strand cDNA synthesis using High Capacity cDNA Reverse Transcription Kit (Applied Biosystems, United states), according to given instructions in the manual. Primer pairs were designed from the untranslated regions (UTRs) of the target genes using Primer Express^®^ (version 3.0) software with the default parameters. For the internal control, elongation factor1-alpha (*ArEF1a*) was used. Each primer combination gave specific amplification of single desired band. Moreover, only one melting temperature was observed for each primer pair while dissociation curve testing. Quantitative real-time PCR (qRT-PCR) was carried out using the PowerUp^TM^ SYBR^®^ Green Master Mix (ABI) in a 7900HT Real-Time PCR System. Each reaction mixture contained 10 μl of SYBR^®^ Green PCR Master Mix, 2 μl of cDNA, and 18 pmol of each primer in a final volume of 20 μl. The thermal cycling parameters were 2 min at 50°C, 10 min at 95°C, and 40 cycles of 15 s at 95°C, and 1 min at 60°C. Each reaction was performed in triplicate. Transcripts of each gene and reference gene *ArEF1a* were amplified using the primers listed in Supplementary Table S1. The obtained values for each gene were then normalized according to the *C*_T_ values of *ArEF1a* (Singh et al., 2012; Nizam et al., 2014b). Relative gene expression levels were calculated using the ΔΔCT method.

## Results

### Genome-Wide Identification and Classification of *A. rabiei* Transcription Factors

**FIGURE 1:**
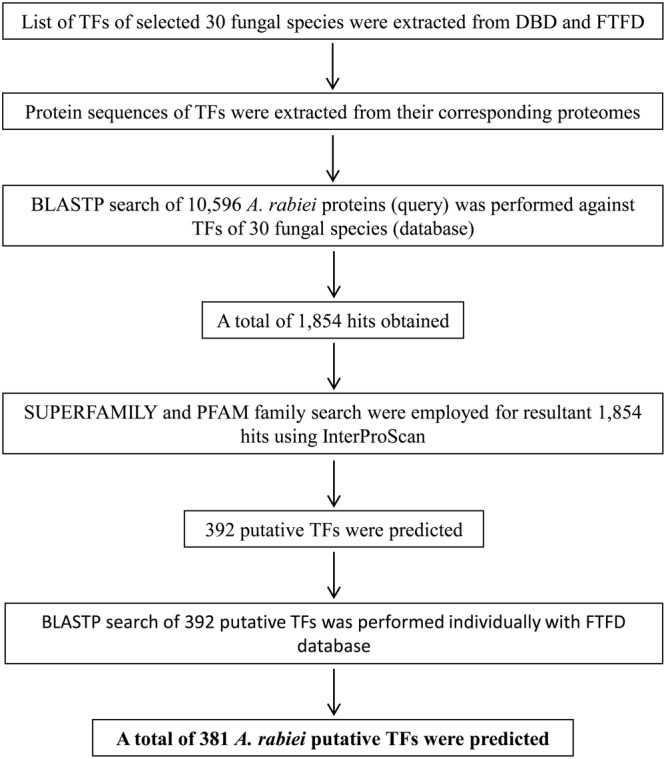
Overview of the computational pipeline used to identify the putative TFs of *A. rabiei*. Based on sequence similarity with characterized TFs from other fungi and the presence of conserved DNA-binding domain regions, proteins were assigned as TFs. A set of 381 putative TFs were predicted in the genome of *A. rabiei*.

There are no bioinformatics tools available to predict the putative TFs in filamentous fungi. Therefore, a systematic workflow was employed in order to identify the putative TFs encoded by *A. rabiei* genome (**Figure 1**). For this, first of all, a comprehensive list of 30 different fungal species was prepared (**Table 1**). These fungal species were selected on the basis of their ecology and lifestyle. The list consisted of 26 fungal species belonging to *Dothideomycetes, Eurotiomycetes, Leotiomycetes, Sordariomycetes, Saccharomycetes*, and *Schizosaccharomycetes* classes of ascomycetes and also 4 fungal species from the phylum basidiomycetes. They represented different forms of lifestyle such as necrotrophy, hemibiotrophy, biotrophy, saprotrophy, symbiosis, and animal pathogens. These criteria were chosen to ensure maximum possible coverage while predicting the putative TFs of *A. rabiei*. The list of predicted TFs of these 30 fungal species was extracted from DBD: a TF prediction database (Wilson et al., 2008) and Fungal Transcription Factor Database (FTFD) (Park et al., 2008). Since these databases provided only the list of TFs and not their protein sequences, thus, the proteomes of the 30 fungal species were downloaded to obtain the corresponding protein sequences. Now these predicted TFs of the 30 fungal species was used as database to predict putative TFs of *A. rabiei*. BLASTP search was carried out using 10,596 protein sequences of *A. rabiei* as query against TFs of 30 species as database. A total of 1,854 hits were obtained. In fungi, 12 superfamilies and 37 PFAM families of TF DBDs are predicted to exist (Shelest, 2008). Considering this, SUPERFAMILY (Gough et al., 2001) and PFAM family (Finn et al., 2014) search were employed on 1,854 resultant hits using InterProScan (Jones et al., 2014). A total of 392 proteins were predicted to have TF DBDs from 12 superfamilies and 37 PFAM families. To minimize false predictions, each of the 392 putative TFs was employed for BLAST search in FTFD database. Sixteen putative TFs that failed to show any match in FTFD database were searched in NCBI database and were selected if they displayed significant homology with any known TF. From these analyses, a set of 381 proteins were obtained with significant hits (Supplementary Dataset S2) and were designated as the putative TFs of *A. rabiei*.

**Table 1:** List of fungal species used in this study.

S. No.	Species	Class; Order	Proteome link	Lifestyle	Predicted putative TFs
1.	*Alternaria brassicicola*	*Dothideomycetes; Pleosporales*	http://genome.jgi.doe.gov/Altbr1/Altbr1.download.ftp.html	Necrotroph	n.a.
2.	*Ashbya gossypii ATCC 10895*	*Saccharomycetes; Saccharomycetales*	http://genome.jgi.doe.gov/pages/dynamicOrganismDownload.jsf?organism=Ashgo1	Biotroph	n.a.
3.	*Aspergillus flavus*	*Eurotiomycetes; Eurotiales*	http://genome.jgi.doe.gov/pages/dynamicOrganismDownload.jsf?organism=Aspfl1	Necrotroph	n.a.
4.	*Aspergillus fumigatus*	*Eurotiomycetes; Eurotiales*	http://genome.jgi.doe.gov/pages/dynamicOrganismDownload.jsf?organism=Aspfu1	Animal pathogen	n.a.
5.	*Blumeria graminis f. sp. hordei DH14*	*Leotiomycetes; Erysiphaceae*	http://genome.jgi.doe.gov/pages/dynamicOrganismDownload.jsf?organism=Blugr1	Biotroph	224
6.	*Botrytis cinerea*	*Leotiomycetes; Helotiales*	http://genome.jgi.doe.gov/pages/dynamicOrganismDownload.jsf?organism=Botci1	Necrotroph	389
7.	*Candida albicans SC5314*	*Saccharomycetes; Saccharomycetales*	http://genome.jgi.doe.gov/pages/dynamicOrganismDownload.jsf?organism=Canalb1	Animal pathogen	n.a.
8.	*Coccidioides immitis*	*Eurotiomycetes; Onygenales*	http://genome.jgi.doe.gov/pages/dynamicOrganismDownload.jsf?organism=Cocim1	Animal pathogen	n.a.
9.	*Cochliobolus heterostrophus*	*Dothideomycetes; Pleosporales*	http://genome.jgi.doe.gov/pages/dynamicOrganismDownload.jsf?organism=CocheC5_3	Necrotroph	362
10.	*Colletotrichum graminicola M1.00*	*Sordariomycetes; Glomerellales*	http://genome.jgi.doe.gov/pages/dynamicOrganismDownload.jsf?organism=Colgr1	Hemibiotroph	n.a.
11.	*Cryptococcus neoformans JEC2*	*Tremellomycetes; Tremellales*	http://genome.jgi.doe.gov/pages/dynamicOrganismDownload.jsf?organism=Cryne_JEC21_1	Animal pathogen	n.a.
12.	*Fusarium graminearu*	*Sordariomycetes; Hypocreales*	http://genome.jgi.doe.gov/pages/dynamicOrganismDownload.jsf?organism=Fusgr1	Necrotroph	n.a.
13.	*Fusarium oxysporum f. sp. lycopersici 4286*	*Sordariomycetes; Hypocreales*	http://genome.jgi.doe.gov/pages/dynamicOrganismDownload.jsf?organism=Fusox1	Hemibiotroph	566
14.	*Fusarium verticillioides 7600*	*Sordariomycetes; Hypocreales*	http://genome.jgi.doe.gov/pages/dynamicOrganismDownload.jsf?organism=Fusve1	Hemibiotroph	n.a.
15.	*Laccaria bicolor*	*Agaricomycetes; Agaricales*	http://genome.jgi.doe.gov/pages/dynamicOrganismDownload.jsf?organism=Lacbi2	Symbiont	613
16.	*Leptosphaeria maculans*	*Dothideomycetes; Pleosporales*	http://genome.jgi.doe.gov/pages/dynamicOrganismDownload.jsf?organism=Lepmu1	Hemibiotroph	n.a.
17.	*Magnaporthe oryzae 70-15*	*Sordariomycetes; Magnaporthales*	http://genome.jgi.doe.gov/pages/dynamicOrganismDownload.jsf?organism=Maggr1	Hemibiotroph	378
18.	*Mycosphaerella fijiensis*	*Dothideomycetes; Capnodiales*	http://genome.jgi.doe.gov/pages/dynamicOrganismDownload.jsf?organism=Mycfi2	Hemibiotroph	n.a.
19.	*Mycosphaerella graminicola*	*Dothideomycetes; Capnodiales*	http://genome.jgi.doe.gov/pages/dynamicOrganismDownload.jsf?organism=Mycgr3	Hemibiotroph	623
20.	*Neosartorya fischeri*	*Eurotiomycetes; Eurotiales*	http://genome.jgi.doe.gov/pages/dynamicOrganismDownload.jsf?organism=Neofi1	Animal pathogen	n.a.
21.	*Neurospora crassa*	*Sordariomycetes; Sordariales*	http://genome.jgi.doe.gov/pages/dynamicOrganismDownload.jsf?organism=Neucr2	Saprotroph	403
22.	*Puccinia graminis f. sp. tritici*	*Pucciniomycetes; Pucciniales*	http://genome.jgi.doe.gov/pages/dynamicOrganismDownload.jsf?organism=Pucgr2	Biotroph	287
23.	*Pyrenophora tritici-repentis*	*Dothideomycetes; Pleosporales*	http://genome.jgi.doe.gov/pages/dynamicOrganismDownload.jsf?organism=Pyrtr1	Necrotroph	366
24.	*Saccharomyces cerevisiae S288C*	*Saccharomycetes; Saccharomycetales*	http://genome.jgi.doe.gov/pages/dynamicOrganismDownload.jsf?organism=Sacce1	Saprotroph	n.a.
25.	*Schizosaccharomyces pombe*	*Schizosaccharomycetes; Schizosaccharomycetales*	http://genome.jgi.doe.gov/pages/dynamicOrganismDownload.jsf?organism=Schpo1	Saprotroph	n.a.
26.	*Sclerotinia sclerotiorum*	*Leotiomycetes; Helotiales*	http://genome.jgi.doe.gov/pages/dynamicOrganismDownload.jsf?organism=Sclsc1	Necrotroph	431
27.	*Parastagonospora nodorum*	*Dothideomycetes; Pleosporales*	http://genome.jgi.doe.gov/pages/dynamicOrganismDownload.jsf?organism=Stano2	Necrotroph	435
28.	*Trichoderma virens*	*Sordariomycetes; Hypocreales*	http://genome.jgi.doe.gov/pages/dynamicOrganismDownload.jsf?organism=TriviGv29_8_2	Saprotroph	n.a.
29.	*Ustilago maydis 521*	*Ustilaginomycetes; Ustilaginales*	http://genome.jgi.doe.gov/pages/dynamicOrganismDownload.jsf?organism=Ustma1	Biotroph	272
30.	*Verticillium dahliae VdLs. 17*	*Sordariomycetes; Glomerellales*	http://genome.jgi.doe.gov/pages/dynamicOrganismDownload.jsf?organism=Verda1	Hemibiotroph	n.a.

*n.a., not analyzed.*

**FIGURE 2:**
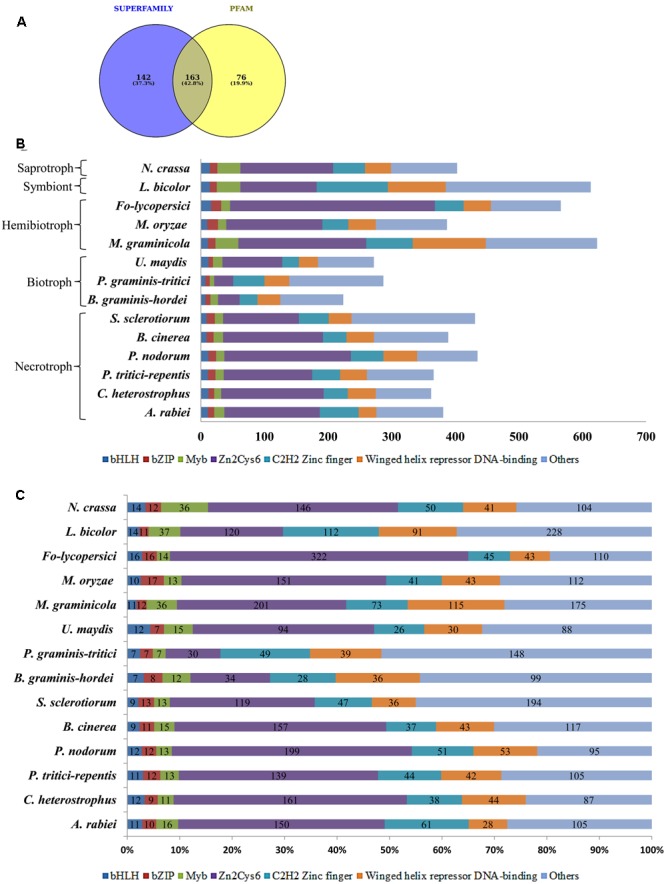
Distribution of *A. rabiei* putative TFs among different structural categories. **(A)** Out of 381 putative TFs predicted, 142 had significant matches with fungi specific 12 superfamilies and 76 had similarities with fungi specific 37 PFAM families (Shelest, 2008). Whereas 163 proteins showed matches in both SUPERFAMILY and PFAM family database. **(B)** The *A. rabiei* putative TFs were compared with TF repertoire of other necrotrophs, biotrophs, hemibiotrophs, symbiont, and saprotroph. The DNA binding domains considered for comparison were winged helix repressor DNA-binding, C_2_H_2_ zinc finger, [Zn(II)_2_Cys_6_], Myb, bZIP, and bHLH domains. The less represented DNA binding domains were categorized under others. **(C)** The relative abundance of each of the selected TF families across the fungal species is shown. The numbers inside the bars of graph are the absolute number of TFs in that family.

Among the 381 putative TFs of *A. rabiei*, 142 and 76 proteins showed significant matches in SUPERFAMILY and PFAM family databases, respectively, whereas 163 proteins were common in both (**Figure 2A**). These 381 putative TFs were then classified and annotated on the basis of 83 InterPro terms present in the InterPro database (Finn et al., 2017), which is also the basis of fungal TF annotation in FTFD database. It grouped 381 *A. rabiei* putative TFs into 32 families (Supplementary Table S2). The maximum number of putative TFs (150) belonged to zinc-cluster superfamily [Zn(II)_2_Cys_6_], which is the largest class of fungal-specific domains. This was followed by C_2_H_2_ type zinc finger domains having 61 putative TFs. The third abundant family of putative TFs in *A. rabiei* was nucleic acid-binding-OB-fold with 45 proteins.

### Comparison of *A. rabiei* Transcription Factors with Other Fungal Species

In order to compare *A. rabiei* putative TF repertoire with other fungal genomes, 13 fungal species were selected representing the major lifestyles. Following the same workflow as for *A. rabiei*, putative TFs were predicted for these 13 fungal species (**Table 1**). The number of predicted TFs in closely related necrotrophic fungi *Cochliobolus heterostrophus* and *Pyrenophora tritici-repentis* were 362 and 366, respectively, similar to the 381 putative TFs of *A. rabiei*. A set of 389 putative TFs were predicted in *Botrytis cinerea*. In *Parastagonospora nodorum* and *Sclerotinia sclerotiorum*, 435 and 431 putative TFs were predicted, respectively. Interestingly, the predicted putative TFs in selected biotrophic fungi, i.e., *Blumeria graminis* f. sp. *hordei* (224), *Puccinia graminis* f. sp. *tritici* (287), and *Ustilago maydis* (272) were comparatively fewer than the number of putative TFs predicted in necrotrophic fungi. Also, the hemibiotrophic fungi *Mycosphaerella graminicola* (623) and *Fusarium oxysporum* f. sp. *lycopersici* (566), and symbiont *Laccaria bicolor* (613) had significantly higher number of predicted TFs. However, the hemibiotrophic fungi *Magnaporthe oryzae* (378) and saprotroph *Neurospora crassa* (403) had putative TFs close to putative TFs predicted for *A. rabiei*.

The predicted TFs from 13 fungal species were classified and annotated on the basis of 83 InterPro terms (Supplementary Table S2). All the fungal species, except *B. graminis* f. sp. *hordei* and *P. graminis* f. sp. *tritici*, had the highest number of putative TFs belonging to zinc-cluster superfamily [Zn(II)_2_Cys_6_]. The HMG (High Mobility Group) and AraC type helix-turn-helix family TFs were abundant in *A. rabiei* similar to *M. graminicola* and *L. bicolor*. The Myb TFs were also more in *A. rabiei* as compared to other necrotrophs. Also, C_2_H_2_ zinc finger TFs were significantly higher in *A. rabiei* unlike most of the selected fungal species, particularly biotrophic fungi (**Figures 2B,C**). The winged helix repressor DNA-binding family was little underrepresented in *A. rabiei* compared to other fungi. This indicates that *A. rabiei* putative TFs are distributed among different classes where the total number of predicted TFs and their distribution into distinct classes differ marginally from those of the selected biotrophic, hemibiotrophic, and symbiotic fungi.

### Gene Structure Analysis of Most Prevalent *A. rabiei* Transcription Factor Families

**FIGURE 3:**
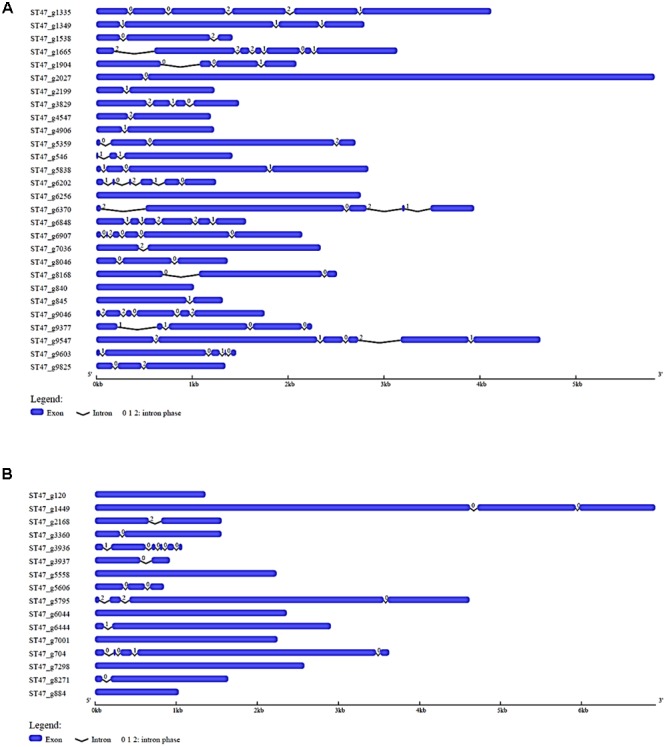
Gene structure analysis. The exon–intron organization is shown for **(A)** winged helix repressor DNA-binding, and **(B)** Myb family of *A. rabiei* putative TFs. Exons and introns are represented by blue rectangles and black lines, respectively. The numbers 0, 1, and 2 represent the intron phase.

To explore the structural diversity of most abundant putative TFs of *A. rabiei*, we analyzed their exon–intron organization. In the most prevalent zinc-cluster superfamily [Zn(II)_2_Cys_6_] having 150 putative TFs, 10 genes were intronless while 28 had single intron (Supplementary Figure S1). The intron phases for all the genes were also analyzed. There are three different phase classes to which introns can be assigned: phase 0, 1, and 2. Phase 0 intron locates between two codons while phase 1 intron splits codons between the first and second nucleotides, and intron is said to be phase 2 when it splits codons between the second and third nucleotides. Half of the single intronic genes encoding zinc-cluster [Zn(II)_2_Cys_6_] TFs had intron phase 0. In C_2_H_2_ zinc finger TFs, 13 genes were equally found intronless and single intronic with phase 1 intron as the most prevalent one (Supplementary Figure S2). The third most abundant putative TF family, nucleic acid-binding-OB-fold, had 6 intronless genes and 13 single intron genes with most of them having intron phase 1 (Supplementary Figure S3). Out of 28 winged helix repressor DNA-binding TFs, 2 were intronless and 6 were single intron genes with phase 1 as the most common intron phase (**Figure 3A**). In Myb TF family, intronless genes were 6 and single intron genes were 5 and had intron phase 0 in most of them (**Figure 3B**). Altogether, this analysis reveals that single intron genes with intron phase either 0 or 1 were predominant in the abundantly found putative TFs of *A. rabiei*.

### Domain Organization and Phylogenetic Analysis of Myb, bHLH, and bZIP Transcription Factor Families

**FIGURE 4:**
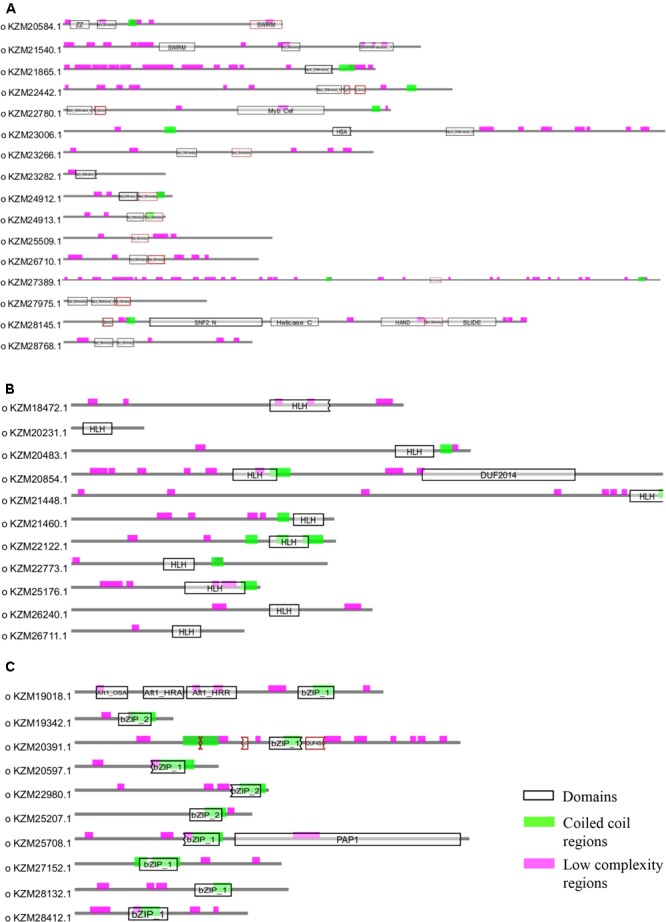
Structural analysis of putative TFs. The protein domains of **(A)** Myb, **(B)** bHLH, and **(C)** bZIP family of *A. rabiei* putative TFs are shown. The domains are denoted by black outlined hollow rectangles, whereas coiled coil regions and low complexity regions are represented by green and pink rectangles, respectively.

Owing to the significant biological functions of TFs, we studied their domain architecture and phylogenetic relationships to gain evolutionary insights. The three families of putative TFs, Myb, bHLH, and bZIP, showed presence of characteristic domains in their protein sequences (**Figure 4**). The Myb TFs had numerous low-complexity regions which are the regions containing little diversity in their amino acid composition (**Figure 4A**). Basic helix-loop-helix domain was present in each of the members of bHLH TFs; however, the size of domain was varying (**Figure 4B**). Similarly, all bZIP TFs of *A. rabiei* had a bZIP domain of uniform size with coiled coil regions overlapping with the bZIP domain (**Figure 4C**). Both these domains are important for the dimerization of the proteins.

**FIGURE 5:**
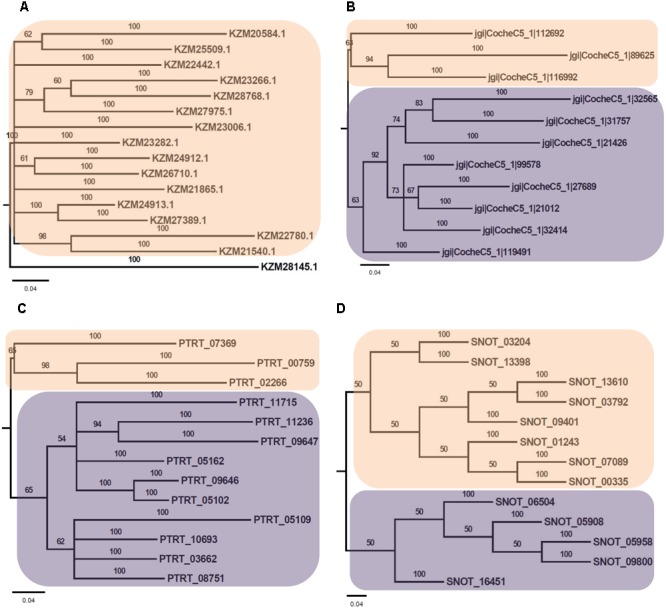
Phylogenetic analysis of Myb family of putative TFs. **(A)** The evolutionary relationship of Myb family of *A. rabiei* putative TFs was compared to that of **(B)** *C. heterostrophus*, **(C)** *P. tritici-repentis*, and **(D)** *P. nodorum* based on Bayesian inference analysis. Each clade is highlighted by colored rectangular block. The Bayesian posterior probabilities are indicated at the nodes.

**FIGURE 6:**
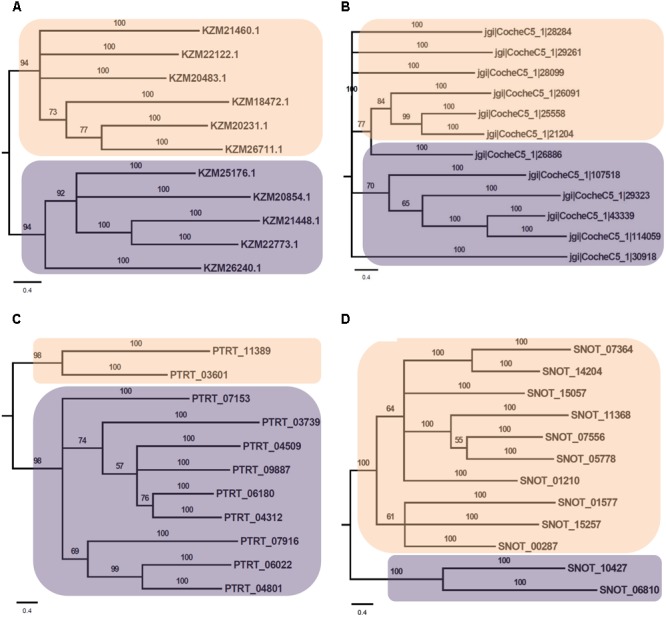
Phylogenetic analysis of bHLH family of putative TFs. **(A)** The evolutionary relationship of bHLH family of *A. rabiei* putative TFs was compared to that of **(B)** *C. heterostrophus*, **(C)** *P. tritici-repentis*, and **(D)** *P. nodorum* based on Bayesian inference analysis. Each clade is highlighted by colored rectangular block. The Bayesian posterior probabilities are indicated at the nodes.

**FIGURE 7:**
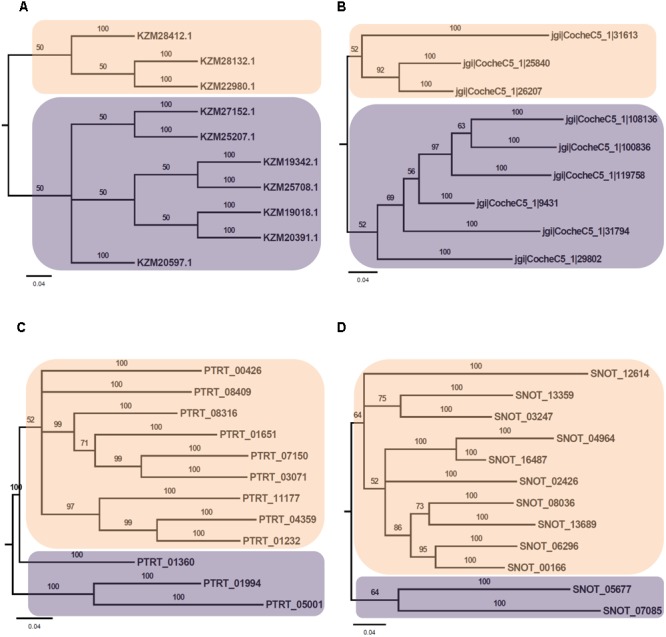
Phylogenetic analysis of bZIP family of putative TFs. **(A)** The evolutionary relationship of bZIP family of *A. rabiei* putative TFs was compared to that of **(B)** *C. heterostrophus*, **(C)** *P. tritici-repentis*, and **(D)** *P. nodorum* based on Bayesian inference analysis. Each clade is highlighted by colored rectangular block. The Bayesian posterior probabilities are indicated at the nodes.

Phylogenetic analysis of Myb TFs of *A. rabiei* with other closely related necrotrophic fungi, i.e., *C. heterostrophus, P. tritici-repentis* and *P. nodorum* was performed. It showed that two major clades were formed in *C. heterostrophus, P. tritici-repentis*, and *P. nodorum* (**Figure 5**). On the contrary, *A. rabiei* had Myb TFs grouped into single clade only. The bHLH TFs showed much conserved distribution in the selected fungal species. They were distributed into two major clades in all of the four fungi (**Figure 6**). Similarly, the bZIP TFs were also divided into two clades (**Figure 7**). The bHLH and bZIP TFs particularly showed very similar pattern of phylogenetic tree between *A. rabiei* and *C. heterostrophus*. In the same way, *P. tritici-repentis* and *P. nodorum* had identical phylogenetic relationship of these TFs. This suggests that these putative TFs have evolved in highly conserved manner in *A. rabiei* and other closely related necrotrophic fungi.

### *Cis*-Regulatory Elements in Promoter of *A. rabiei* Genes Encoding Secretory Proteins

**FIGURE 8:**
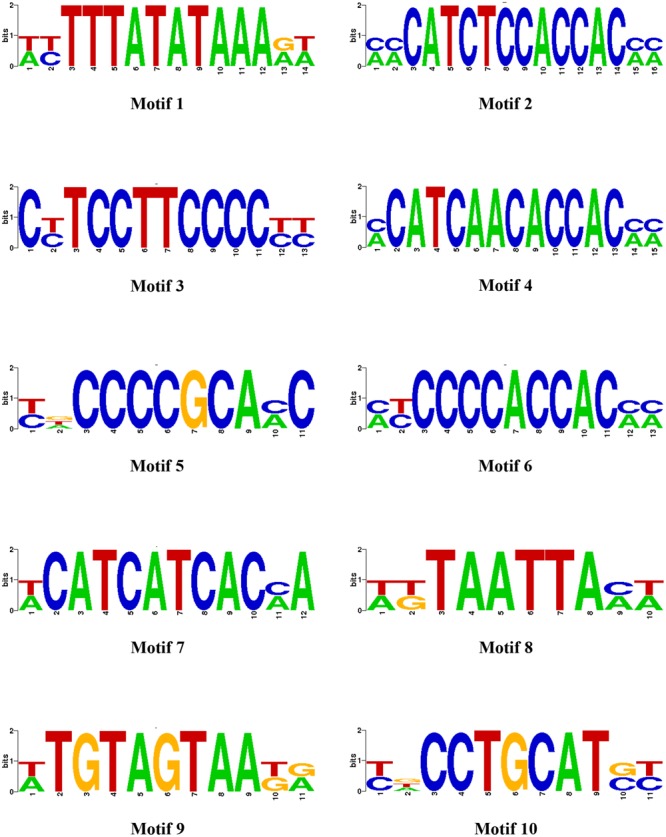
The most enriched *cis*-regulatory elements in the promoter sequences of secretory protein coding genes. Ten most abundant motifs in the promoter sequences of genes encoding *A. rabiei* secretory proteins are shown, as identified by RSAT suite for fungi (Regulatory Sequence Analysis).

We earlier reported the necrotrophic effector repertoire of *A. rabiei* (Verma et al., 2016). A set of 758 putative secretory proteins were predicted to constitute the secretome. Therefore, we investigated the *cis*-regulatory elements present in the promoter sequences of those *A. rabiei* genes that encode secretory proteins. This would aid in identifying the putative TFs that regulate the co-ordinated expression of *A. rabiei* secretome. For this, up to 1 kb 5′ flanking sequence of the genes encoding putative secretory proteins were selected and any other gene sequences occurring in these promoter regions were discarded. A scan in promoters of the 758 genes was performed using RSAT suite (Medina-Rivera et al., 2015) to obtain highly frequent DNA patterns known to be the binding sites of TFs. After scanning, the 10 most frequent motifs were identified (**Figure 8** and **Table 2**). The motif CATCAACACCAC was the most recurrent motif that was predicted to bind to the promoter regions of 234 genes out of 758 genes encoding secretory proteins and had 316 numbers of occurrences. This was followed by CATCTCCACCAC motif that was identified in the promoters of 222 genes with 303 instances of occurrences. The third most abundant motif was TCCTTCCCC, which was present at 216 promoter sequences and had 273 matches.

**Table 2:** Ten most enriched *cis*-regulatory elements identified in the promoter sequences of secretory protein coding genes.

Name	Motifs	UniProbe Database match	*E*-value (UniProbe match)	Matches	No. of promoters
Motif_1	wyTTTATATAAArw	Nhp6a (HMG factors)	0.000002	303	123
Motif_2	mmCaTCTCCACCACmm	Cha4 (C6 zinc cluster factors)	0.004379	303	222
Motif_3	cyTCCTTCCCcyy	Rap1 (Myb)	0.21617	273	216
Motif_4	mcATCAACACCACmm	Rap1 (Myb)	0.015551	316	234
Motif_5	ydCCCCGCAmc	YML081W (zf-C2H2)	0.000008	200	167
Motif_6	myCCCCACCACmm	RPN4 (zf-C2H2)	0.003615	243	184
Motif_7	wcATCATCACma	Pbf2 (Myb)	0.002092	273	205
Motif_8	wkTAATTAmw	Yox1 (Homeobox)	0	164	71
Motif_9	wtGTAGTAAkr	Sko1 (bZIP_1)	0.008315	214	156
Motif_10	ydCCTGCATsy	Phd1 (HTH APSES-type)	0.000388	157	141

Once we identified the most frequent *cis*-regulatory elements across the promoter regions of the genes encoding secretory proteins of *A. rabiei*, the TFs known to bind on these sites were identified. For this, the motifs were scanned for their corresponding TFs using UniPROBE database (Newburger and Bulyk, 2009). It revealed that Myb TFs were the corresponding TFs that bind to CATCAACACCAC motif (**Table 2**). For the second most abundant motif CATCTCCACCAC, [Zn(II)_2_Cys_6_] zinc cluster TFs were predicted to bind. The Myb TFs were also predicted to bind to the motif TCCTTCCCC. Among the 10 most frequent motifs identified, the corresponding TFs for 3 motifs were Myb TFs. In addition, two *cis*-regulatory elements were regulated by C_2_H_2_ zinc finger TFs. Likewise, the [Zn(II)_2_Cys_6_] zinc cluster, bZIP, APSES, HMG, and homeobox TFs were predicted to bind on 1 of the 10 motifs identified. This suggests that the secretome of *A. rabiei* is regulated by an array of TFs, mainly Myb TFs.

### Differentially Expressed Transcription Factor Genes of *A. rabiei* during Host Infection

**FIGURE 9:**
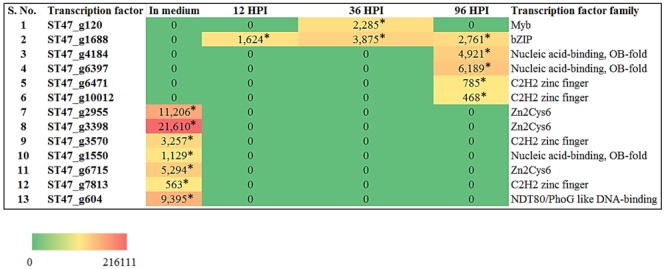
Heat map representation of *A. rabiei* putative TFs. The expression of *A. rabiei* putative TF genes is shown during vegetative growth (in medium) and host invasion [12, 36, and 96 hours post inoculation (hpi)]. For this, *A. rabiei* transcriptome data was utilized (Fondevilla et al., 2015). Expression of putative TFs genes are denoted in the terms of FPKM values. Values with asterisk represent *P*-value < 0.005 at which differentially expressed genes (DEGs) were found.

Recently, the transcriptome data of *A. rabiei* during plant invasion was reported (Fondevilla et al., 2015). We utilized this data in order to identify the differentially expressed genes (DEGs) encoding predicted TFs during host infection. The RNA-seq reads of “*in medium*” and “*in planta*” samples at time points 12, 36, and 96 hpi were mapped on *A. rabiei* genome and expression values were calculated in the terms of FPKM. A total of 13 putative TF encoding genes were found differentially expressed (**Figure 9**). Interestingly, seven genes were found to be expressed exclusively *in medium* and were not at all expressed during *in planta* conditions (**Figure 9**). Likewise six genes which were expressed during *in planta* conditions had no expression *in medium*. Four putative TF encoding genes were found expressed exclusively at 96 hpi whereas only one putative TF (bZIP) encoding gene was expressing at all the three time points during plant infection (**Figure 9**). The DEGs mainly belonged to [Zn(II)_2_Cys_6_] zinc cluster, C_2_H_2_ zinc finger and nucleic acid-binding-OB-fold family of TFs.

**FIGURE 10:**
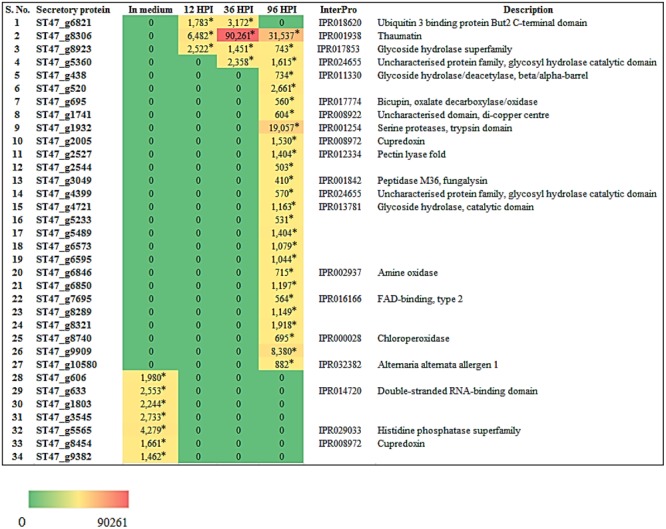
Heat map representation of *A. rabiei* putative secretory protein coding genes. The expression pattern of genes encoding *A. rabiei* putative secretory proteins is shown during vegetative growth (in medium) and host invasion (12, 36, and 96 hpi). For this, *A. rabiei* transcriptome data was utilized (Fondevilla et al., 2015). Expression of putative secretory protein coding genes are denoted in the terms of FPKM values. Values with asterisk represent *P*-value < 0.05 at which DEGs were found.

We also investigated the expression of genes encoding secretory proteins of *A. rabiei* that we have predicted earlier (Verma et al., 2016). A set of 34 putative secretory protein coding genes were found differentially expressed (**Figure 10**). During *in planta* conditions, 27 genes showed differential expression at different time points and these genes were not expressed when the fungus was grown in *in vitro* condition. At 96 hpi, most of the secretory protein coding genes (26) were up-regulated and out of these, 23 genes were exclusively expressed at this time point of infection. On the other hand, two secretory protein coding genes were differentially expressed at all the three time points of infection. Interestingly, there were seven genes which were expressed only during *in medium* condition. This suggests that *A. rabiei* exhibits a much orchestrated spatial and temporal distribution of secretory proteins during its lifecycle.

In order to get the insights of putative TFs that regulate the secretory proteins of *A. rabiei*, we searched for the presence of most frequent *cis*-regulatory elements in the promoters of secretory protein encoding genes which were found up-regulated during *in medium* and *in planta* conditions. Twelve secretory proteins had CATCAACACCAC motif in their promoters and this motif is the binding site for Myb TFs, particularly Rap1 (Supplementary Dataset S3). Similarly, CCTGCAT motif was found in promoters of 11 secretory proteins and is the binding site of APSES TF (Phd1). The bZIP and C_2_H_2_ zinc finger TFs appeared to regulate expression of 7 secretory proteins due to the presence of their binding sites on promoter sequences. It indicates that an array of putative TFs regulate secretory proteins during host invasion and *in vitro* condition.

### Expression Analysis of *A. rabiei* Transcription Factors by qRT-PCR

**FIGURE 11:**
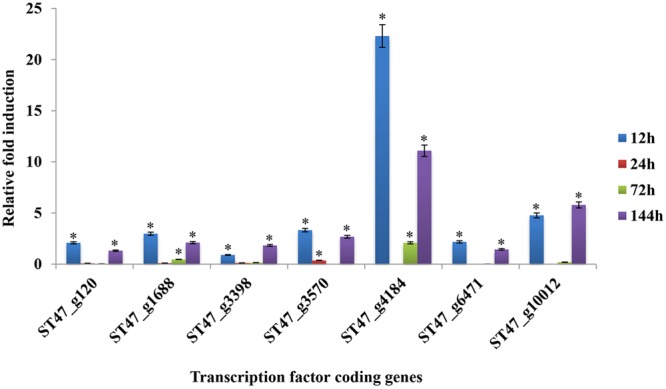
Expression profiles of putative TF genes assayed by qRT-PCR during host colonization. Bar diagrams representing the expression pattern of seven putative TF genes are shown as the fold-change compared to the control. Expression was analyzed in *A. rabiei*-infected chickpea samples at 12, 24, 72, and 144 hpi. *ArEF1a* gene was used as an internal reference gene. Asterisks denote significant difference compared with the expression level at 0 hpi (*p* < 0.05).

For transcriptomic studies of *A. rabiei*–chickpea pathosystem (Fondevilla et al., 2015), *A. rabiei* isolate P4 (originated from Kaljebrin, Syria) was used to inoculate susceptible chickpea cultivar “Blanco Lechoso.” In our laboratory, we use ArD2 isolate of *A. rabiei* (obtained from Indian Agricultural Research Institute, New Delhi, India) representing pathotype 2 to infect highly susceptible chickpea cultivar Pusa-362. Therefore, we investigated whether the putative TFs exhibit similar expression pattern in *A. rabiei*–chickpea pathosystem when the pathotype of *A. rabiei* and the chickpea cultivar were different. For this, the expression profile of seven different putative TFs during host colonization was assayed by qRT-PCR at 12, 24, 72, and 144 hpi (**Figure 11**). All the selected genes were found differentially expressed during infection. In comparison with other putative TF genes, the abundance of *ST47_g4184* transcripts (nucleic acid-binding-OB-fold) was highest at almost all the time points. Its expression was highest at 12 hpi that drastically reduced at 24 hpi and then again gradually increased at 72 and 144 hpi suggesting a bi-phasic induction. Similarly, *ST47_g10012* (C_2_H_2_ zinc finger) also showed maximum expression at 12 and 144 hpi. In terms of expression pattern, the transcripts of *ST47_g120* (Myb), *ST47_g1688* (bZIP), *ST47_g3570* (C2H2 zinc finger), *ST47_g4184* and *ST47_g6471* (C2H2 zinc finger) showed maximum expression at 12 hpi followed by 144 hpi suggesting that these genes were getting induced during initial phases of pathogenesis. While *ST47_g3398* [Zn(II)_2_Cys_6_] and *ST47_g10012* appeared to play role during later stages of pathogenesis as they showed maximum transcript abundance at 144 hpi. Overall, these results indicate that TFs may exhibit differential expression in a temporal manner specific to the pathotype of *A. rabiei* and the cultivar of chickpea.

## Discussion

Whenever a pathogen attacks host plant, extensive reprogramming of gene expression facilitated by TFs occur in both the host and pathogen (Verma et al., 2013). Several distinct reports suggest the crucial role played by TFs in the growth, development and virulence of filamentous fungi. Consequently, the attention has been recently focused to study TFs since they regulate an array of pathogenicity and stress responsive genes. In yeast and filamentous fungi, a basic leucine zipper (bZIP) TF Activating Protein 1 (AP-1) acts as a transcriptional activator in response to oxidative stress (Wu and Moye-Rowley, 1994; Toone and Jones, 1998; Reverberi et al., 2008) and carries out multiple functions as a redox regulator (Fernandes et al., 1997; Karin et al., 1997). The yeast AP-1 family of TFs consists of Yap1 in *S. cerevisiae*, Pap1 in *S. pombe*, Cap1 in *Candida albicans*, and Kap1 in *Kluyveromyces lactis* (Toone et al., 2001). Later, Yap1 orthologs were also identified in *Aspergillus nidulans, Aspergillus fumigatus*, and *Aspergillus parasiticus* where they act as key players in cellular defense against oxidative stress (Asano et al., 2007; Reverberi et al., 2008, 2012). However, in necrotrophic phytopathogenic fungus *B. cinerea*, a bZIP TF (BcAtf1) regulates expression of catalase B but does not contribute to osmotic and oxidative stress tolerance (Temme et al., 2012). In *M. oryzae*, MST12, a homolog of the yeast TF Ste12, plays crucial role in host penetration and colonization (Park et al., 2002). Few other specific TFs, for instance *Cochliobolus carbonum* ccSNF1, regulates *in planta* expression of cell wall degrading enzymes (Tonukari et al., 2000). In *Fusarium graminearum*, the pathogenicity and sexual development is largely affected by a Myb-like transcription factor MYT3 (Kim et al., 2014). Similarly, the AbPf2 TF of *Alternaria brassicicola* is an important regulator of pathogenesis and it does not affect other cellular processes (Cho et al., 2013). Therefore, TFs are vital for survival and completion of the lifecycle.

*Ascochyta* spp. are highly devastating pathogens causing severe losses in production of legumes worldwide. However, not much is known about the pathogenicity mechanisms of this necrotrophic fungus. There are only few reports available which are not quite sufficient to shed light on understanding of *A. rabiei*–chickpea pathosystem. Despite extensive pathological studies, the nature and extent of pathogenic variability in *A. rabiei* have not been clearly established. The mechanisms by which *A. rabiei* infects and colonizes chickpea plants remain poorly understood. Though, with the recent advances in genomics, transcriptomics, and molecular studies of *A. rabiei*, it is now possible to carry out in depth investigations of *A. rabiei* pathogenicity. The genetic manipulations/transformations of *A. rabiei* have made identification of gene functions possible (Nizam et al., 2010). Similarly, various comprehensive studies of *A. rabiei* gene families have been performed in order to gain the molecular and evolutionary insights (Nizam et al., 2014a,b; Kim et al., 2015).

With the present study, we have further expanded the understanding of *A. rabiei* as a pathogen. Since TFs have a major role in fungal development, pathogenesis and response to the environment, we have identified and classified the putative TF repertoire of *A. rabiei*. This detailed analysis was achievable mainly due to the availability of *A. rabiei* genome sequencing data. A comprehensive computational approach was implemented that resulted in identification of a set of 381 proteins as the putative TFs of *A. rabiei*. In fungi, usually the TFs comprise 0.5–8% of the gene content and can be classified on the basis of structure of their DBDs (Stegmaier et al., 2004; Wilson et al., 2008). In *A. rabiei*, TFs constitute 3.5% of the gene content. Among the *A. rabiei* putative TFs predicted, [Zn(II)_2_Cys_6_] family of TFs was overwhelmingly predominated. The [Zn(II)_2_Cys_6_] TFs are usually the most abundant TFs in fungi as revealed by few studies analyzing 37 known fungal transcription regulator-related PFAM domains in several ascomycete and basidiomycete genomes (Shelest, 2008; Todd et al., 2014). A variety of cellular and metabolic processes are regulated by this class of TFs. In model filamentous ascomycete fungus *Podospora anserina*, the two zinc cluster proteins RSE2 and RSE3 regulates expression of genes encoding alternative oxidase, phosphoenolpyruvate carboxykinase, fructose-1,6-biphosphatase, alternative NADH dehydrogenase, a [Zn(II)_2_Cys_6_] TF, a flavohemoglobin, and various hydrolases (Bovier et al., 2014). Necrotrophic fungus *B. cinerea* has a [Zn(II)_2_Cys_6_] TF, BcGaaR that induces D-galacturonic acid (GalA)-inducible genes and promotes growth of *B. cinerea* on GalA (Zhang et al., 2015). In *F. graminearum*, [Zn(II)_2_Cys_6_] TF EBR1 regulates virulence and apical dominance of the hyphal tip (Zhao et al., 2011). Furthermore, a high-throughput gene knockout of 104 [Zn(II)_2_Cys_6_] TF genes in *M. oryzae* was performed that suggested their significance in growth, asexual and infection-related development, pathogenesis and response to nine abiotic stresses (Lu et al., 2014). It also revealed that [Zn(II)_2_Cys_6_] TFs involved in pathogenicity frequently tend to function in multiple development stages. During hemibiotrophic *Colletotrichum lindemuthianum*-bean interaction, CLTA1 (a fungal zinc cluster TF) regulates biotrophic phase specific genes facilitating the biotrophy to necrotrophy switch (Dufresne et al., 2000). Therefore, the prevalence of [Zn(II)_2_Cys_6_] family of TFs in *A. rabiei* suggests that they might play important role in survival and virulence of *A. rabiei*.

In *A. rabiei*, the C_2_H_2_ zinc finger class was second most abundant class which represents a smaller but major regulator class in the ascomycetes. These are involved in development (Kwon et al., 2010) and calcium signaling in *A. nidulans* (Hagiwara et al., 2008). Another C_2_H_2_ TF, MtfA, is a regulator of secondary metabolism and morphogenesis in *A. nidulans* (Ramamoorthy et al., 2013). Moreover, a conserved C_2_H_2_ TF (RsrA) coordinates a NapA mediated oxidative response in *Aspergillus* fungi (Bok et al., 2014). In *A. fumigatus*, GipA induces production of immunosuppressive secondary metabolite gliotoxin (Schoberle et al., 2014). In *A. parasiticus* and *Aspergillus flavus*, deletion of *MSNA* resulted in enhanced production of conidia, ROS, aflatoxin, and kojic acid (Roze et al., 2004). In addition, the C_2_H_2_ TFs may use multiple recognition motifs to control gene expression (Han et al., 2016). This indicates that C_2_H_2_ zinc finger class of TFs in *A. rabiei* might be the major contributors in regulating the growth and developmental processes.

Comparison of *A. rabiei* putative TFs with that of other necrotrophic, biotrophic, hemibiotrophic, symbiotic, and saprotrophic fungi suggested a conserved as well as unique distribution of TFs among different classes in all the selected fungi. This indicates toward the evolutionary specificity of TFs depending on the lifestyle and host of the fungi. Similar indications were also provided when phylogenetic analysis of *A. rabiei* Myb, bHLH and bZIP TF families was compared with other closely related necrotrophic fungi, i.e., *C. heterostrophus, P. tritici-repentis*, and *P. nodorum*. It suggested formation of well conserved clades in these TF families among the closely related necrotrophic fungi. The domain organization of Myb, bHLH, and bZIP TF families of *A. rabiei* showed presence of characteristic domains as well as numerous low-complexity regions. Various studies have suggested that these regions show significant divergence across protein families. Proteins containing low-complexity regions have more binding partners across different protein–protein interaction networks (Coletta et al., 2010). The low-complexity regions positioned at center of protein sequence are related to transcription-related gene ontology terms, whereas terminally located low-complexity regions are associated with translation and stress response-related terms (Coletta et al., 2010).

The TFs of bZIP family of *A. rabiei* had bZIP domains superimposed with coiled coil regions. Several TFs of bHLH and Myb family also had coiled coil regions. The architecture of a particular coiled-coil domain governs its oligomerization state, rigidity and ability to function as a molecular recognition system. In fungi, Myb TFs play an important role in cell differentiation, development and pathogenicity (Arratia-Quijada et al., 2012; Kim et al., 2014). Similarly, basic helix-loop-helix (bHLH) TFs are also known to be involved in development (Murre et al., 1994). The highly functionally conserved bZIP TFs ensure proper growth, development, sulfur metabolism, and iron homeostasis in fungi (Guo et al., 2011; Kong et al., 2015; Sun et al., 2016). They are highly stress responsive and are vital for pathogenicity in a reactive oxygen species-dependent manner. The gene structure of [Zn(II)_2_Cys_6_], C_2_H_2_, nucleic acid-binding-OB-fold, winged helix repressor DNA-binding and Myb family of TFs suggested that the genes with single intron had intron phase either 0 or 1 predominantly. The three classes of intron phases are far from even distribution. Phase 0 introns are usually present in excess (Long et al., 1995) and the inferred evolution of intron phase distribution showed that the proportion of phase 0 introns increased over evolution (Nguyen et al., 2006). Therefore, the excess of phase 0 introns in *A. rabiei* putative TF genes indicates toward the evolutionary development of this pathogen over the years.

Earlier, we predicted a set of 758 proteins in the secretome of *A. rabiei* (Verma et al., 2016). Since the secretory proteins are largely involved in the pathogenesis and deriving nutrition from the host, the *cis*-regulatory elements present in promoter sequences of secretory proteins coding genes of *A. rabiei* were identified. The secretome coding genes of *A. rabiei* were predicted to be significantly regulated by Myb TFs followed by [Zn(II)_2_Cys_6_] zinc cluster TFs. Fungal genomes have at least 37 PFAM families of TF (Shelest, 2008). So far, 12 TFs from four families (Zinc finger, APSES, WOPR, and Fork head) have been found to regulate the gene expression of candidate effectors (Tan and Oliver, 2017). A central role for Zn-finger TF in effector expression was studied in *A. brassicicola* where a [Zn(II)_2_Cys_6_] zinc cluster TF (AbPf2) regulates expression of 106 genes, out of which 33 genes encode secreted proteins, including eight putative effector proteins (Cho et al., 2013). Plants challenged with *Δabpf2* mutants had elevated expression levels of photosynthesis, pentose phosphate pathway and primary metabolism related genes but decreased levels of defense-related genes. In *P. nodorum*, AbPf2 ortholog PnPf2 positively regulates the necrotrophic effectors *SnToxA* and *SnTox3* expression (Rybak et al., 2017). Likewise, PtrPf2 controls the expression of *P. tritici-repentis ToxA*, a near-identical copy of *SnToxA* (Rybak et al., 2017). This indicates that Pf2 TF exhibits a conserved role in regulating the effectors. Furthermore, SnStuA (an APSES bHLH TF) was found to regulate the expression of *SnTox3* (IpCho et al., 2010). In *Leptosphaeria maculans*, the expression of *AvrLm4-7, AvrLm1*, and *AvrLm6* is regulated by LmStuA (Soyer et al., 2015). The example of WOPR TFs was found in *Verticillium dahlia* and *Cladosporium fulvum*. The VdSge1 TF is required for full expression of the Cys-rich effector *Ave1* (Santhanam and Thomma, 2013). Similarly, CfWor1 TF primarily regulates development of *Cladosporium*, but also indirectly controls expression of a subset of effector genes (Ökmen et al., 2014). In *U. maydis*, a fork head TF named *Fox1* acts as a positive regulator of six candidate effector genes (Zahiri et al., 2010). Over all, these studies indicate the significance of TFs in regulating the effectors that ultimately govern the pathogenesis. The *A. rabiei* secretome was predicted to be mainly regulated by Myb TFs, therefore, it would be highly significant to identify the potential Myb TFs that controls the expression of *A. rabiei* effectors.

In order to understand significance of *A. rabiei* secretome and putative TFs during host colonization, the RNA-seq data of *A. rabiei* during *in medium* and *in planta* conditions were analyzed (Fondevilla et al., 2015). Thirty-four secretory protein coding genes and seven putative TF genes were differentially expressed. Most of these secretory protein coding genes had binding sites of Myb TFs (particularly Rap1) in their promoter sequences. This is consistent with our computational prediction suggesting Myb TFs as the major regulators of *A. rabiei* secretome. The qRT-PCR analysis of few *A. rabiei* putative TFs showed a lot of differences in expression profiling as compared to RNA-seq data. The putative TFs ST47_3398 and ST47_3570, which were not expressing during *in planta* conditions in RNA-seq data, showed expression in qRT-PCR analysis. Furthermore, RNA-seq data showed that majority of putative TFs were expressed during later stages of infection. By contrast, according to qRT-PCR analysis almost all selected putative TFs were expressed in early stages of infection as well. Biphasic kinetics of gene expression is commonly observed in both pathogen and host during infection (Schneider et al., 2011; Singh et al., 2012). This suggests that the expression profile of these putative TFs could be differential depending on the pathotype of *A. rabiei* and the cultivar of chickpea. Further investigation in this direction is a prerequisite to determine if TFs function discretely in different pathotypes of *A. rabiei*.

Since TFs as regulators play a crucial role in the life cycle of fungi, their presence or absence may offer opportunities or enforce limitations on the natural habitat of fungal species. The present study provides a platform to study *A. rabiei* TFs in detail. This will offer better insight into the evolution of regulatory mechanisms in *A. rabiei*. Comparative studies of *A. rabiei* TFs in other pathotypes will decipher their contribution in determining the pathotypes of this fungus. The extensive knowledge obtained will aid in designing successful strategies to control this devastating pathogen and prevent further crop losses.

## Author Contributions

SV, RG, and PV designed the experiments; RG and SV performed bioinformatics analysis; SV performed the experiments; SV, RG, and PV analyzed data; and SV, RG, and PV wrote the manuscript.

## Conflict of Interest Statement

The authors declare that the research was conducted in the absence of any commercial or financial relationships that could be construed as a potential conflict of interest.

## Footnotes

1 http://www.transcriptionfactor.org

2 http://ftfd.snu.ac.kr/

3 http://www.ebi.ac.uk/Tools/pfa/iprscan5/

4 http://gsds.cbi.pku.edu.cn

5 http://cdvist.utk.edu

6 http://www.ebi.ac.uk/Tools/msa/muscle/

7 http://tree.bio.ed.ac.uk/software/figtree/

8 http://www.rsat.eu/

9 http://thebrain.bwh.harvard.edu/uniprobe/
