# Supplementary material for: Transcription Factor Repertoire of Necrotrophic Fungal Phytopathogen Ascochyta rabiei: Predominance of MYB Transcription Factors As Potential Regulators of Secretome
Source: Front Plant Sci. 2017 Jun 14;8:1037. doi: 10.3389/fpls.2017.01037 (PMC5470089; doi:10.3389/fpls.2017.01037)
Supplement: Supplementary file 1 [file Table_1.PDF]

Supplementary Material

Transcription Factor Repertoire of Necrotrophic Fungal Phytopathogen *Ascochyta rabiei*: Predominance of MYB Transcription Factors as Potential Regulators of Secretome

Sandhya Verma[1#], Rajesh Kumar Gazara[1#] and Praveen Kumar Verma[1*]

[1]Plant Immunity Laboratory, National Institute of Plant Genome Research, New Delhi, India

[#]Authors contributed equally to this work

[*]Correspondence: Dr. Praveen Kumar Verma: pkv@nipgr.ac.in

Supplementary Table S1 | List of primers used to amplify genes needed for gene expression analyses.

Primer name	Forward primer (5'-3')	Reverse primer (5'-3')
ST47_g120 RT	ACGCGTTCCAAGTTTCACCTT	GGGCTTGAGACAAAACAATGACA
ST47_g1688 RT	GAGGCCAGCTGTCCAGTTTG	GGGTGGAGTTGGGTGCAA
ST47_g3398 RT	CCGCACTCCACAGCTTGAC	TGCAGGCTAGTGCTCTTGTAAAGA
ST47_g3570 RT	CGCCATGTGTTGCCGTATC	GACATAACCGCACTTTGATGCTAT
ST47_g4184 RT	CTGCTGCATGCCATTCTTTC	CGAGCGTGCGGTTAGTTCTC
ST47_g6471 RT	ATGGCAGTTGTGTCGTTGATTT	AGGTGGACATTTCGAAAACAATG
ST47_g10012 RT	CGTTCCGCAGCCTCATCTT	CCAACCCAAGGGTGAAGGA
