# Supplementary material for: Transcription Factor Repertoire of Necrotrophic Fungal Phytopathogen Ascochyta rabiei: Predominance of MYB Transcription Factors As Potential Regulators of Secretome
Source: Front Plant Sci. 2017 Jun 14;8:1037. doi: 10.3389/fpls.2017.01037 (PMC5470089; doi:10.3389/fpls.2017.01037)
Supplement: Supplementary file 2 [file Table_2.PDF]

Supplementary Material

Transcription Factor Repertoire of Necrotrophic Fungal Phytopathogen *Ascochyta rabiei*: Predominance of MYB Transcription Factors as Potential Regulators of Secretome

Sandhya Verma[1#], Rajesh Kumar Gazara[1#] and Praveen Kumar Verma[1*]

[1]Plant Immunity Laboratory, National Institute of Plant Genome Research, New Delhi, India

[#]Authors contributed equally to this work

[*]**Correspondence:** Dr. Praveen Kumar Verma: pkv@nipgr.ac.in

Supplementary Table S2 | Genes encoding TFs in *A. rabiei* and other fungi along with their distribution among different families of fungal TFs.

S. No.	Family	Ar	Ch	Ptr	Sn	Bc	Ss	Bgh	Pgt	Um	Mg	Mo	Fol	Lb	Nc
1	APSES	4	4	5	4	5	5	4	4	4	4	5	5	5	5
2	AT-rich interaction region	-	1	1	1	2	2	2	-	1	3	2	-	3	2
3	bHLH	11	12	11	12	9	9	7	7	12	11	10	16	14	14
4	bZIP	10	9	12	12	11	13	8	7	7	12	17	16	11	12
5	CCR4-Not complex component, Not1	1	1	1	1	1	1	1	1	1	1	1	1	1	1
6	Grainyhead/ CP2	1	1	1	1	1	1	1	-	-	1	1	1	-	2
7	Cold-shock protein, DNA-binding	-	-	-	-	-	-	-	3	-	-	-	1	-	-
8	DDT	1	1	1	1	1	1	1	-	1	3	1	1	3	1
9	SGT1	1	1	1	1	1	1	1	1	1	1	1	1	1	1
10	Forkhead	4	3	5	4	4	4	4	2	5	6	4	4	4	4
11	HMG	4	1	2	1	1	1	1	-	-	11	1	-	16	1
12	Centromere protein B, DNA-binding region	1	-	2	1	8	56	1	2	1	2	3	2	5	3
13	Helix-turn-helix type 3	2	2	1	2	1	1	1	1	-	-	-	1	1	1
14	Helix-turn-helix, AraC type	6	-	-	-	-	-	-	-	-	22	-	-	40	-
15	Helix-turn-helix, Psq	-	1	-	-	1	33	-	-	1	-	-	-	-	1
16	Heteromeric CCAAT factors	2	1	1	1	1	1	1	1	1	10	1	1	6	1
17	Homeobox	5	9	10	12	7	7	4	9	6	4	7	12	11	7
18	Homeodomain -like	2	4	7	3	10	9	7	34	7	3	4	7	15	3
19	Lambda repressor-like, DNA-binding	-	-	1	-	1	1	-	1	1	2	1	1	1	2
20	Mating-type	-	-	1	1	1	1	-	-	-	1	-	1	-	2

	protein MAT alpha 1														
21	Myb	16	11	13	13	15	13	12	7	15	36	13	14	37	20
22	NDT80/PhoG like DNA-binding	2	-	-	-	-	-	-	-	-	-	-	-	-	-
23	Negative transcriptional regulator	1	1	-	-	2	1	-	-	1	-	1	2	-	-
24	Nucleic acid-binding, OB-fold	45	37	40	35	42	41	44	34	30	52	43	41	45	47
25	SART1	1	1	1	1	1	1	1	2	1	1	1	1	1	1
26	TEA/ATTS	1	-	1	1	1	1	1	1	1	1	-	1	6	1
27	MADS-box	2	2	2	2	1	3	2	2	2	3	2	2	3	2
28	Transcription factor jumonji	1	-	-	-	-	-	-	1	-	2	-	1	5	-
29	Tubby transcription factors	1	-	-	-	-	-	-	-	-	-	-	-	-	-
30	Winged helix repressor DNA-binding	28	44	42	53	43	36	36	39	30	115	43	43	91	41
31	YL1 nuclear protein	1	1	1	1	1	1	1	--	1	1	1	1	1	1
32	Zinc finger, BED-type	-	-	-	-	-	-	-	25	-	-	-	-	-	-
33	Zinc finger, C2H2	61	38	44	51	37	47	28	49	26	73	41	45	112	50
34	Zinc finger, CCHC-type	7	1	7	8	4	5	7	9	4	11	5	5	18	6
35	Zinc finger, DHHC-type	-	-	-	-	-	-	-	-	-	6	-	-	7	-
36	Zinc finger, GATA-type	7	7	6	7	7	7	6	6	11	12	8	8	16	8
37	Zinc finger, GRF-type	-	-	-	-	3	2	1	1	2	1	2	2	-	4
38	Zinc finger, MIZ-type	1	2	2	2	2	1	2	3	1	1	2	1	2	3
39	Zinc finger, NF-X1-type	-	1	1	-	1	1	1	-	-	1	1	1	1	1
40	Zinc finger, PARP-type	-	2	2	1	1	1	1	1	1	1	1	1	2	1
41	Zinc finger, Rad18-type putative	-	-	-	-	-	-	-	-	-	1	-	-	-	-
42	Transcription	-	-	-	-	-	-	-	-	-	3	-	-	4	-

	factor TFIIS														
43	Zn2Cys6	150	161	139	199	157	119	34	30	94	201	151	322	120	146
44	Copper fist DNA binding domain-containing protein	1	-	-	1	2	1	-	1	-	1	1	1	2	1
45	p53-like transcription factor	-	2	2	2	3	3	3	3	3	2	3	3	2	7
46	ssDNA-binding transcriptional regulator	-	-	-	-	-	-	-	-	-	1	-	-	1	-
	**Total**	**381**	**362**	**366**	**435**	**389**	**431**	**224**	**287**	**272**	**623**	**378**	**566**	**613**	**403**

Ar: *A. rabiei*, **Ch:** *Cochliobolus heterostrophus*, **Ptr:** *Pyrenophora tritici-repentis*, **Sn:** *Stagonospora nodorum*, **Bc:** *Botrytis cinerea*, **Ss:** *Sclerotinia sclerotiorum*, **Bgh:** *Blumeria graminis* f. sp. *hordei*, **Pgt:** *Puccinia graminis* f. sp. *tritici*, **Um:** *Ustilago maydis*, **Mg:** *Mycosphaerella graminicola*, **Mo:** *Magnaporthe oryzae*, **Fol:** *Fusarium oxysporum* f. sp. *lycopersici*, **Lb:** *Laccaria bicolor*, **Nc:** *Neurospora crassa*.
