# Supplementary material for: Transcription Factor Repertoire of Necrotrophic Fungal Phytopathogen Ascochyta rabiei: Predominance of MYB Transcription Factors As Potential Regulators of Secretome
Source: Front Plant Sci. 2017 Jun 14;8:1037. doi: 10.3389/fpls.2017.01037 (PMC5470089; doi:10.3389/fpls.2017.01037)
Supplement: Supplementary file 6 [file Image_1.PDF]

Supplementary Material

Transcription Factor Repertoire of Necrotrophic Fungal Phytopathogen *Ascochyta rabiei*: Predominance of MYB Transcription Factors as Potential Regulators of Secretome

Sandhya Verma[1#], Rajesh Kumar Gazara[1#] and Praveen Kumar Verma[1*]

[1]Plant Immunity Laboratory, National Institute of Plant Genome Research, New Delhi, India

[#]Authors contributed equally to this work

[*]Correspondence: Dr. Praveen Kumar Verma: pkv@nipgr.ac.in

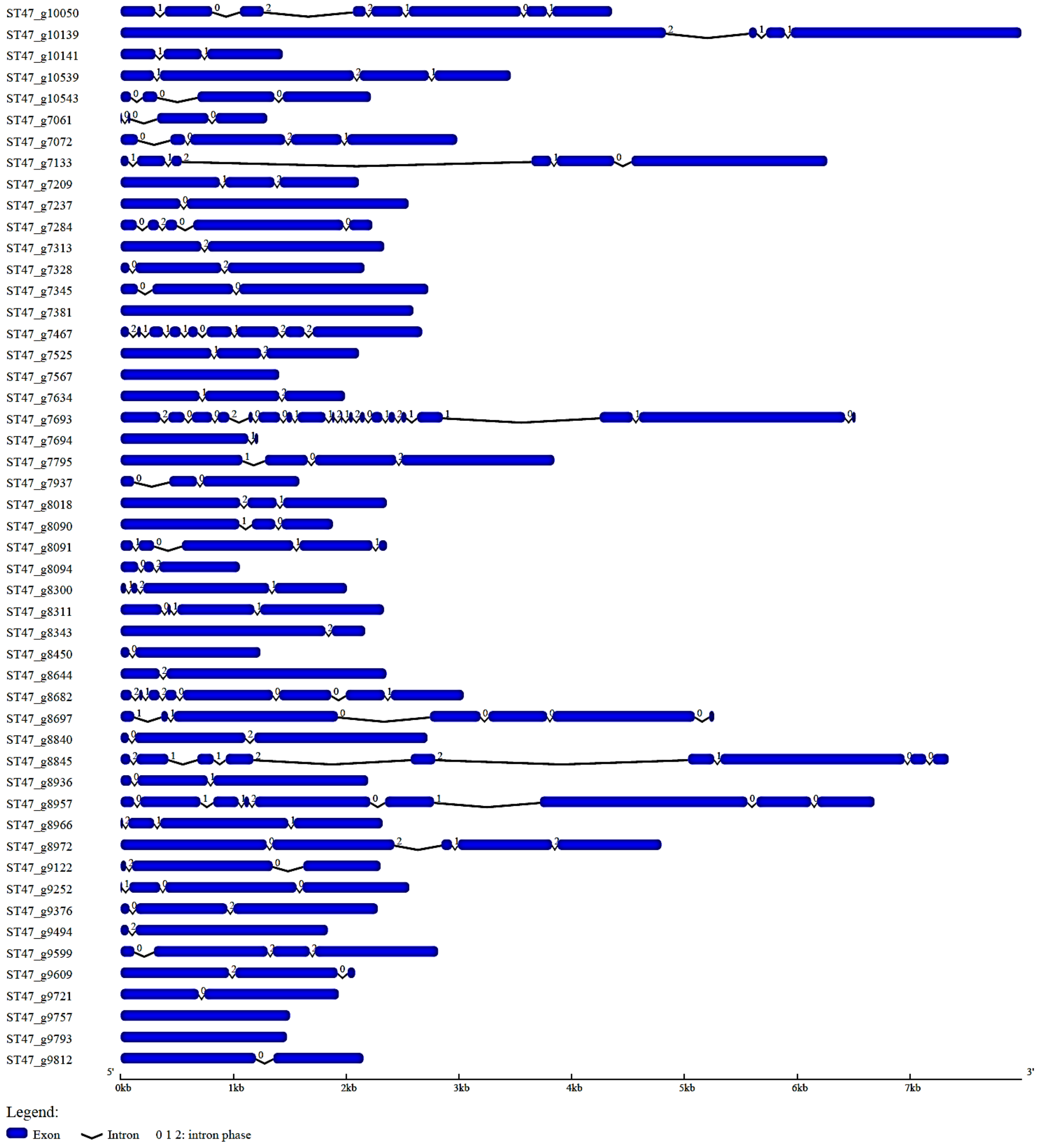
ST47_g10050
ST47_g10139
ST47_g10141
ST47_g10539
ST47_g10543
ST47_g7061
ST47_g7072
ST47_g7133
ST47_g7209
ST47_g7237
ST47_g7284
ST47_g7313
ST47_g7328
ST47_g7345
ST47_g7381
ST47_g7467
ST47_g7525
ST47_g7567
ST47_g7634
ST47_g7693
ST47_g7694
ST47_g7795
ST47_g7937
ST47_g8018
ST47_g8090
ST47_g8091
ST47_g8094
ST47_g8300
ST47_g8311
ST47_g8343
ST47_g8450
ST47_g8644
ST47_g8682
ST47_g8697
ST47_g8840
ST47_g8845
ST47_g8936
ST47_g8957
ST47_g8966
ST47_g8972
ST47_g9122
ST47_g9252
ST47_g9376
ST47_g9494
ST47_g9599
ST47_g9609
ST47_g9721
ST47_g9757
ST47_g9793
ST47_g9812
5'
3'
0kb
1kb
2kb
3kb
4kb
5kb
6kb
7kb
Legend:
Exon
Intron
0 1 2: intron phase

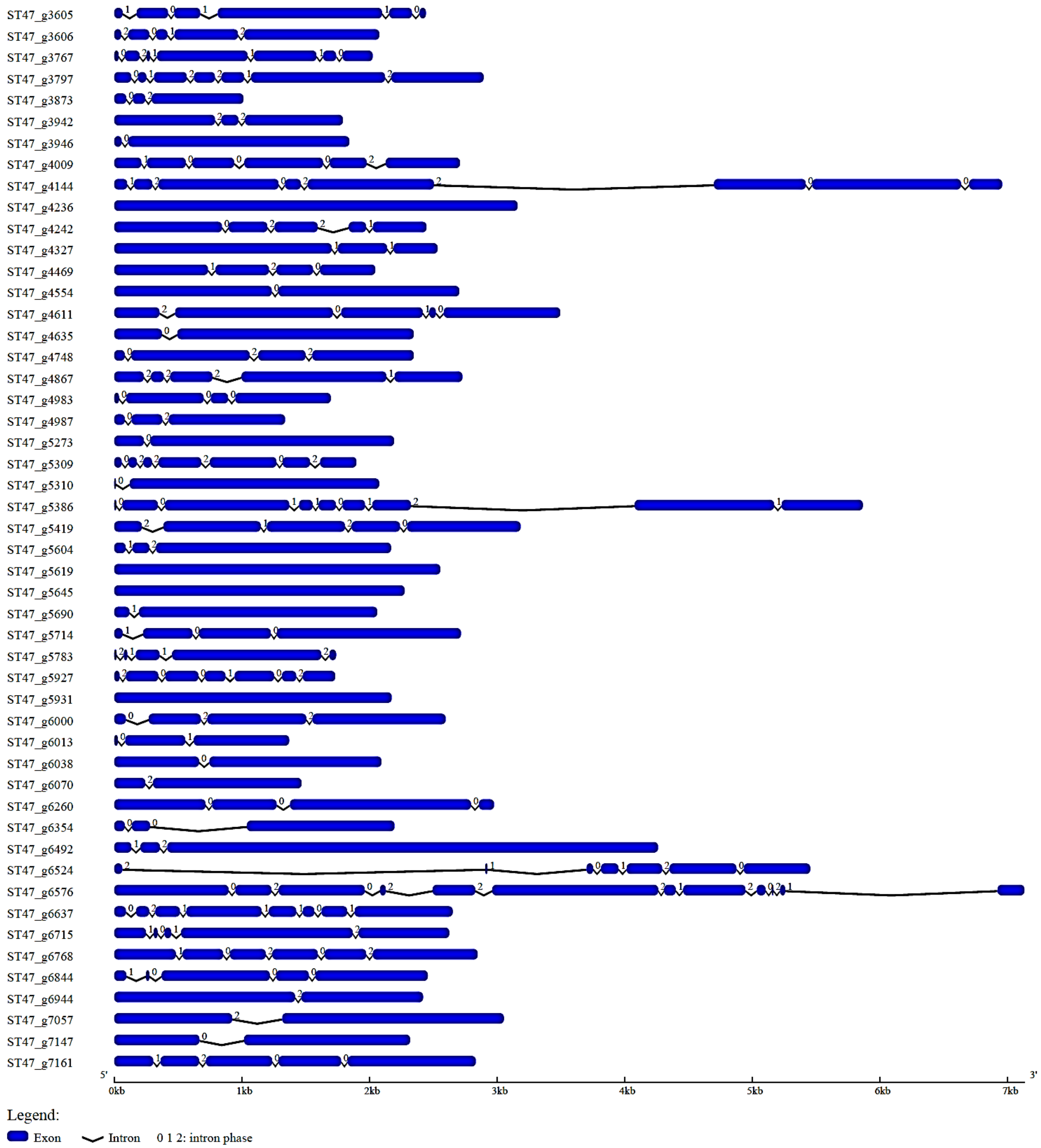
ST47_g3605
ST47_g3606
ST47_g3767
ST47_g3797
ST47_g3873
ST47_g3942
ST47_g3946
ST47_g4009
ST47_g4144
ST47_g4236
ST47_g4242
ST47_g4327
ST47_g4469
ST47_g4554
ST47_g4611
ST47_g4635
ST47_g4748
ST47_g4867
ST47_g4983
ST47_g4987
ST47_g5273
ST47_g5309
ST47_g5310
ST47_g5386
ST47_g5419
ST47_g5604
ST47_g5619
ST47_g5645
ST47_g5690
ST47_g5714
ST47_g5783
ST47_g5927
ST47_g5931
ST47_g6000
ST47_g6013
ST47_g6038
ST47_g6070
ST47_g6260
ST47_g6354
ST47_g6492
ST47_g6524
ST47_g6576
ST47_g6637
ST47_g6715
ST47_g6768
ST47_g6844
ST47_g6944
ST47_g7057
ST47_g7147
ST47_g7161
5'
3'
0kb
1kb
2kb
3kb
4kb
5kb
6kb
7kb
Legend:
Exon
Intron
0 1 2: intron phase

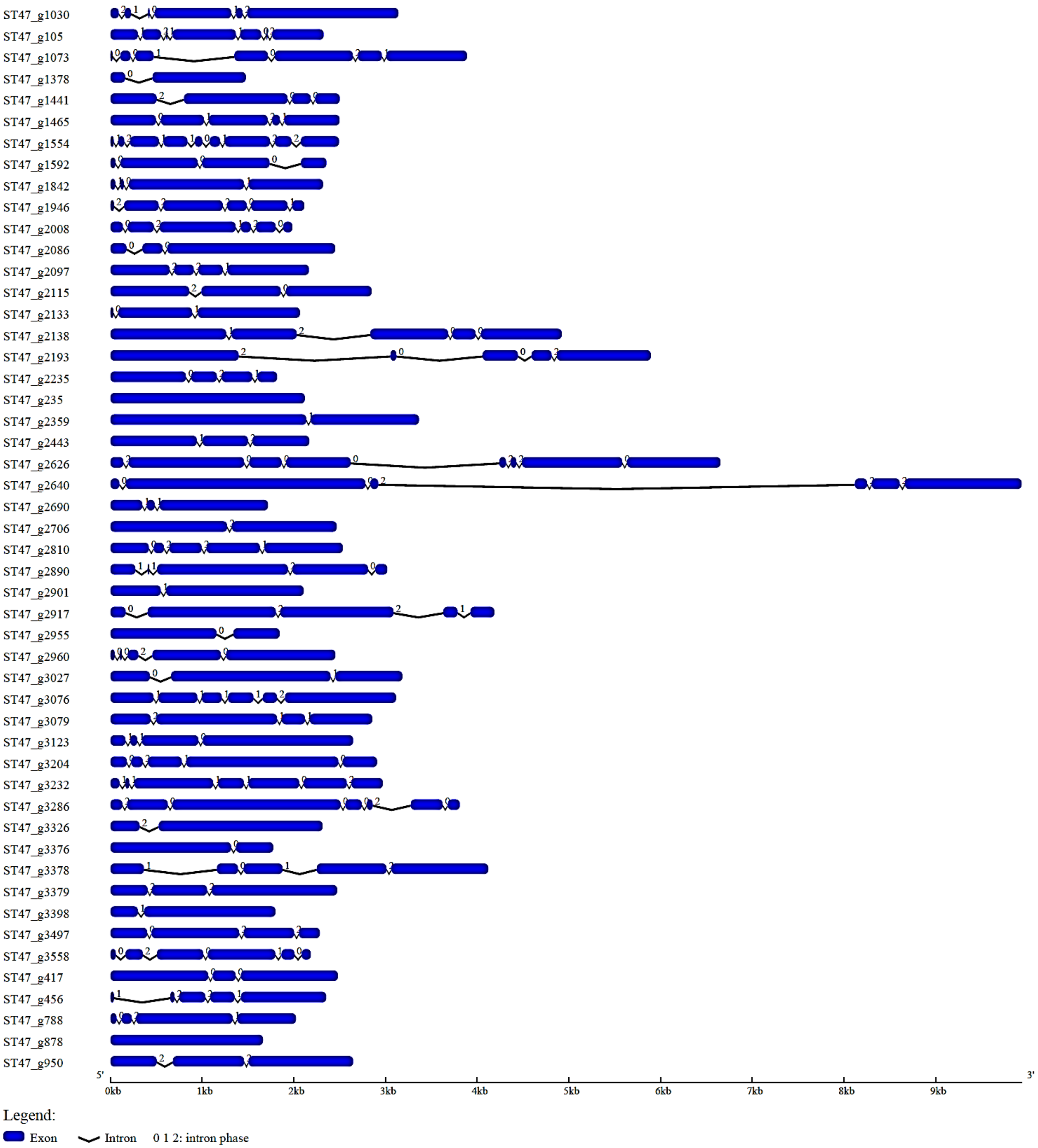

Supplementary Figure S1. Gene structure analysis. The exon-intron organization is shown for $[Zn(II)_2Cys_6]$ family of *A. rabiei* transcription factors. Exons and introns are represented by blue rectangles and black lines, respectively. The numbers 0, 1 and 2 represent the intron phase.
