# Supplementary material for: Transcription Factor Repertoire of Necrotrophic Fungal Phytopathogen Ascochyta rabiei: Predominance of MYB Transcription Factors As Potential Regulators of Secretome
Source: Front Plant Sci. 2017 Jun 14;8:1037. doi: 10.3389/fpls.2017.01037 (PMC5470089; doi:10.3389/fpls.2017.01037)
Supplement: Supplementary file 7 [file Image_2.PDF]

Supplementary Material

Transcription Factor Repertoire of Necrotrophic Fungal Phytopathogen *Ascochyta rabiei*: Predominance of MYB Transcription Factors as Potential Regulators of Secretome

Sandhya Verma[1#], Rajesh Kumar Gazara[1#] and Praveen Kumar Verma[1*]

[1]Plant Immunity Laboratory, National Institute of Plant Genome Research, New Delhi, India

[#]Authors contributed equally to this work

[*]Correspondence: Dr. Praveen Kumar Verma: pkv@nipgr.ac.in

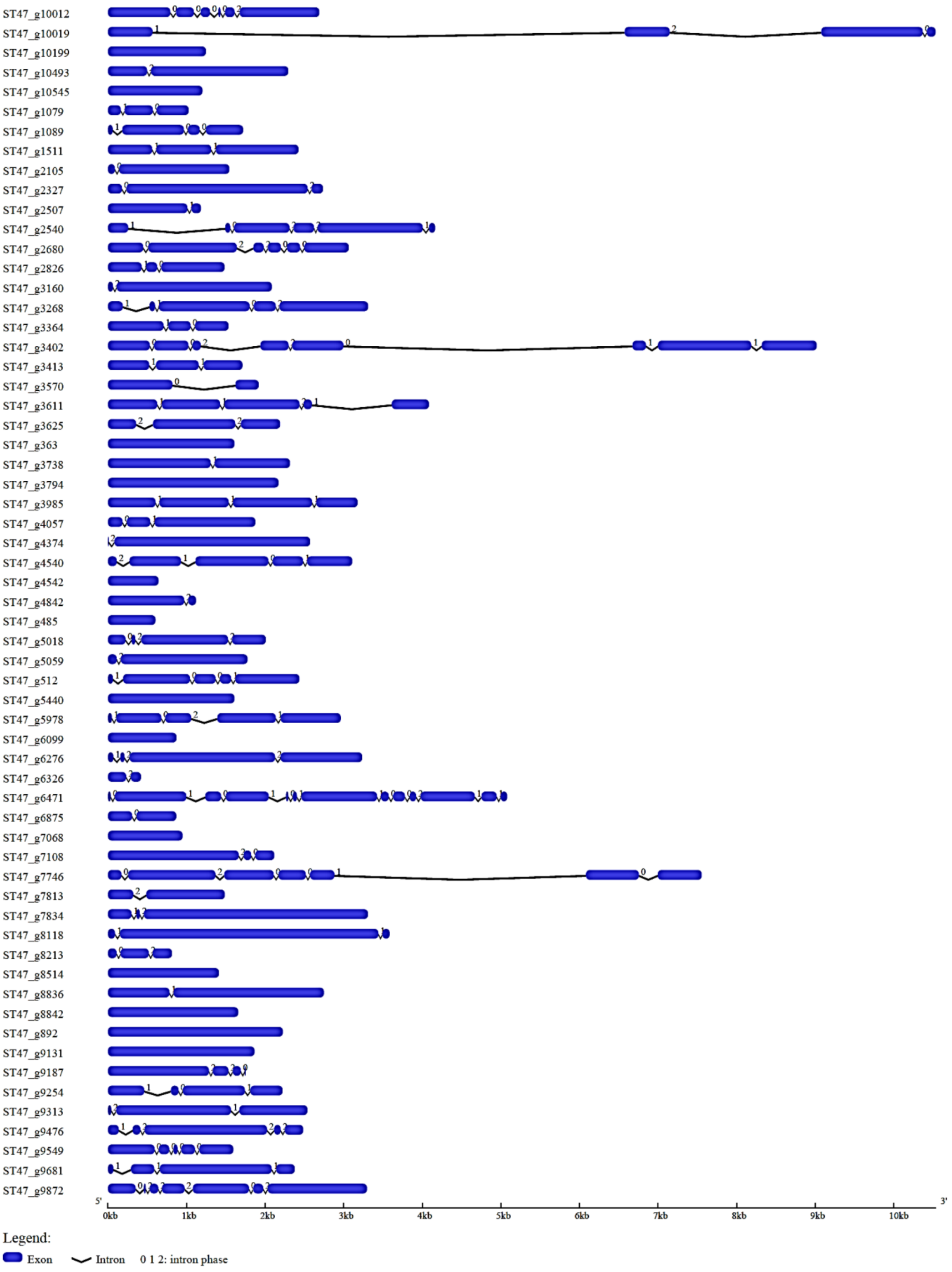
ST47_g10012
ST47_g10019
ST47_g10199
ST47_g10493
ST47_g10545
ST47_g1079
ST47_g1089
ST47_g1511
ST47_g2105
ST47_g2327
ST47_g2507
ST47_g2540
ST47_g2680
ST47_g2826
ST47_g3160
ST47_g3268
ST47_g3364
ST47_g3402
ST47_g3413
ST47_g3570
ST47_g3611
ST47_g3625
ST47_g363
ST47_g3738
ST47_g3794
ST47_g3985
ST47_g4057
ST47_g4374
ST47_g4540
ST47_g4542
ST47_g4842
ST47_g485
ST47_g5018
ST47_g5059
ST47_g512
ST47_g5440
ST47_g5978
ST47_g6099
ST47_g6276
ST47_g6326
ST47_g6471
ST47_g6875
ST47_g7068
ST47_g7108
ST47_g7746
ST47_g7813
ST47_g7834
ST47_g8118
ST47_g8213
ST47_g8514
ST47_g8836
ST47_g8842
ST47_g892
ST47_g9131
ST47_g9187
ST47_g9254
ST47_g9313
ST47_g9476
ST47_g9549
ST47_g9681
ST47_g9872
5'
3'
0kb
1kb
2kb
3kb
4kb
5kb
6kb
7kb
8kb
9kb
10kb
Legend:
Exon
Intron
0 1 2: intron phase

11

Supplementary Figure S2. Gene structure analysis. The exon-intron organization is shown for C_2H_2 family of *A. rabiei* transcription factors. Exons and introns are represented by blue rectangles and black lines, respectively. The numbers 0, 1 and 2 represent the intron phase.
