# Supplementary material for: Transcription Factor Repertoire of Necrotrophic Fungal Phytopathogen Ascochyta rabiei: Predominance of MYB Transcription Factors As Potential Regulators of Secretome
Source: Front Plant Sci. 2017 Jun 14;8:1037. doi: 10.3389/fpls.2017.01037 (PMC5470089; doi:10.3389/fpls.2017.01037)
Supplement: Supplementary file 8 [file Image_3.PDF]

frontiers

Supplementary Material

Transcription Factor Repertoire of Necrotrophic Fungal Phytopathogen *Ascochyta rabiei*: Predominance of MYB Transcription Factors as Potential Regulators of Secretome

Sandhya Verma[1#], Rajesh Kumar Gazara[1#] and Praveen Kumar Verma[1*]

[1]Plant Immunity Laboratory, National Institute of Plant Genome Research, New Delhi, India

[#]Authors contributed equally to this work

[*]Correspondence: Dr. Praveen Kumar Verma: pkv@nipgr.ac.in

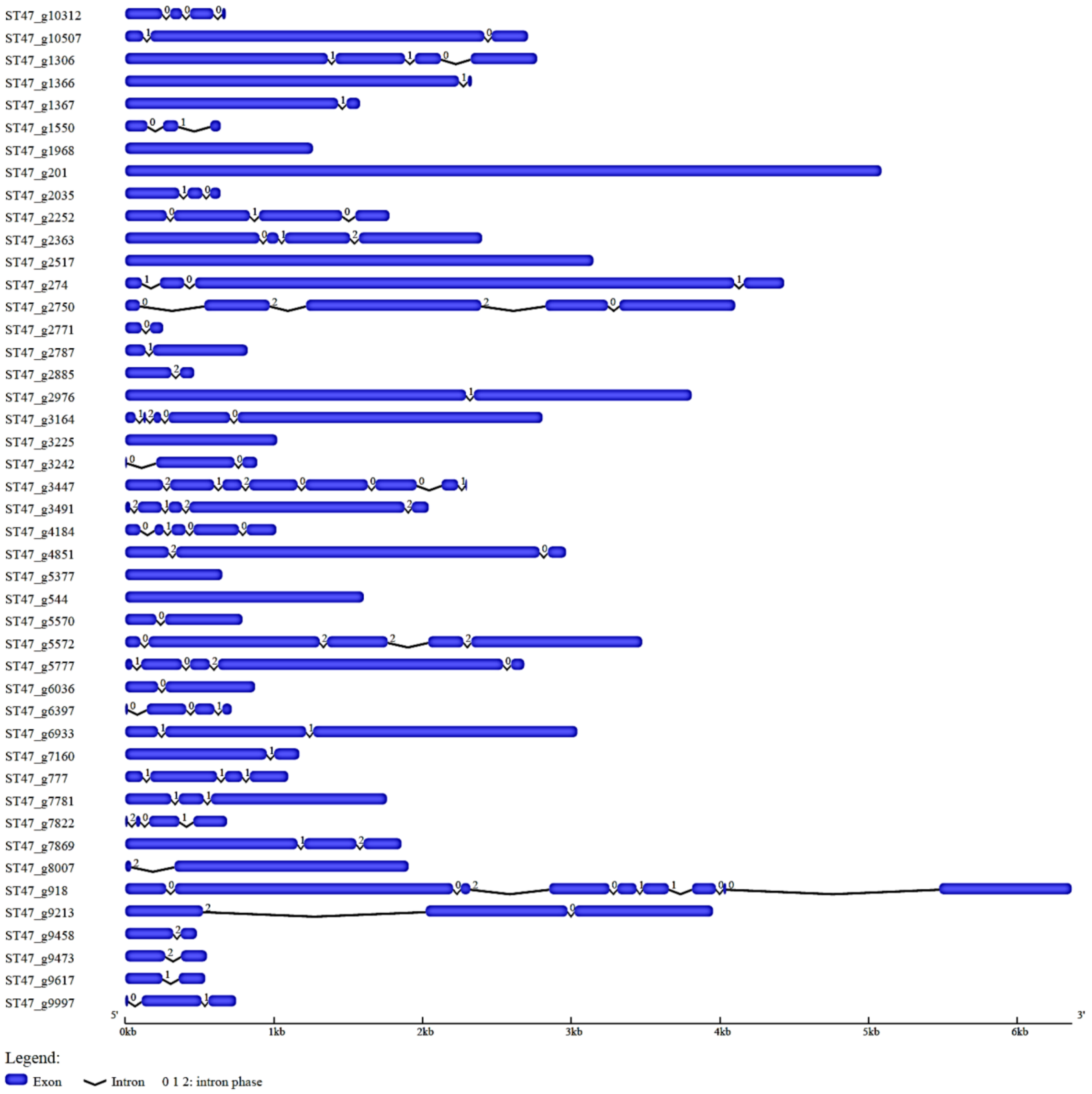

Supplementary Figure S3. Gene structure analysis. The exon-intron organization is shown for Nucleic acid-binding-OB-fold family of *A. rabiei* transcription factors. Exons and introns are represented by blue rectangles and black lines, respectively. The numbers 0, 1 and 2 represent the intron phase.
